# Genome features of *Pseudomonas putida* LS46, a novel polyhydroxyalkanoate producer and its comparison with other *P. putida* strains

**DOI:** 10.1186/s13568-014-0037-8

**Published:** 2014-05-22

**Authors:** Parveen K Sharma, Jilagamazhi Fu, Xiangli Zhang, Brian Fristensky, Richard Sparling, David B Levin

**Affiliations:** 1 Department of Biosystems Engineering, University of Manitoba, Winnipeg R3T 2N2, MB, Canada; 2 Department of Plant Science, University of Manitoba, Winnipeg R3T 2N2, MB, Canada; 3 Department of Microbiology, University of Manitoba, Winnipeg R3T 2N2, MB, Canada

**Keywords:** Pseudomonas putida, Comparative bioinformatics analysis, Comparative genome analysis, Polyhydroxyalkanoates, Pan-Genome, Insertion sequences, Metabolic diversity

## Abstract

A novel strain of *Pseudomonas putida* LS46 was isolated from wastewater on the basis of its ability to synthesize medium chain-length polyhydroxyalkanoates (mcl-PHAs). *P.putida* LS46 was differentiated from other *P.putida* strains on the basis of cpn60 (UT). The complete genome of *P.putida* LS46 was sequenced and annotated. Its chromosome is 5,86,2556 bp in size with GC ratio of 61.69. It is encoding 5316 genes, including 7 rRNA genes and 76 tRNA genes. Nucleotide sequence data of the complete *P. putida* LS46 genome was compared with nine other *P. putida* strains (KT2440, F1, BIRD-1, S16, ND6, DOT-T1E, UW4, W619 and GB-1) identified either as biocontrol agents or as bioremediation agents and isolated from different geographical region and different environment. BLASTn analysis of whole genome sequences of the ten *P. putida* strains revealed nucleotide sequence identities of 86.54 to 97.52%. *P.putida* genome arrangement was LS46 highly similar to *P.putida* BIRD1 and *P.putida* ND6 but was markedly different than *P.putida* DOT-T1E, *P.putida* UW4 and *P.putida* W619. Fatty acid biosynthesis (*fab*), fatty acid degradation (*fad*) and PHA synthesis genes were highly conserved among biocontrol and bioremediation *P.putida* strains. Six genes in *pha* operon of *P. putida* LS46 showed >98% homology at gene and proteins level. It appears that polyhydroxyalkanoate (PHA) synthesis is an intrinsic property of *P. putida* and was not affected by its geographic origin. However, all strains, including *P. putida* LS46, were different from one another on the basis of house keeping genes, and presence of plasmid, prophages, insertion sequence elements and genomic islands. While *P. putida* LS46 was not selected for plant growth promotion or bioremediation capacity, its genome also encoded genes for root colonization, pyoverdine synthesis, oxidative stress (present in other soil isolates), degradation of aromatic compounds, heavy metal resistance and nicotinic acid degradation, manganese (Mn II) oxidation. Genes for toluene or naphthalene degradation found in the genomes of *P. putida* F1, DOT-T1E, and ND6 were absent in the *P. putida* LS46 genome. Heavy metal resistant genes encoded by the *P. putida* W619 genome were also not present in the *P. putida* LS46 genome. Despite the overall similarity among genome of *P.putida* strains isolated for different applications and from different geographical location a number of differences were observed in genome arrangement, occurrence of transposon, genomic islands and prophage. It appears that *P.putida* strains had a common ancestor and by acquiring some specific genes by horizontal gene transfer it differed from other related strains.

## Introduction

The genus *Pseudomonas* consists of a very heterogeneous group of microorganisms isolated from diverse environments and belonging to the gamma (γ)-proteobacteria (Palleroni [1984]). The genus has approximately 100 named species grouped into different subgroups based on multi-locus analysis (Winsor et al. [2011]). The genus *Pseudomonas* contains human pathogens, plant pathogens, and heterotrophic bacteria prevalent in soil, water, and on plant surfaces. Most pseudomonads are free-living saprophytic organisms in soil or water. Because of their great metabolic diversity they play an important role in decomposition, biodegradation, and the carbon and nitrogen cycles. *Pseudomonas putida* is non-pathogenic soil bacteria and is capable of degrading xenobiotic and promoting plant growth after root colonization as well as simultaneously providing protection for the plant from pests and other harmful bacteria (Compant et al. [2005]; Singleton [1994]).

*Pseudomonas putida* KT2440, *P. putida* BIRD1, *P. putida* UW4, *P. putida* S11 were isolated from rhizosphere soil and developed as plant growth promoting rhizobacteria (Glick [1995]; Matilla et al. [2011]; Nakazawa [2002]; Ponraj et al. [2012]). *Pseudomonas putida* F1 was isolated from polluted soil and developed as a bioremediation agent (Choi et al. [2003]; Eaton [1997]). *Pseudomonas putida* S16 was isolated in China and identified as a nicotine degrading strain (Tang et al. [2012]). *Pseudomonas putida* ND6 was identified as a naphthalene degrading bacterium, in which naphthalene degradation genes were located on a plasmid (Dennis et al. [2004]; Li et al. [2002]). *Pseudomonas putida* DOT-T1E was isolated as a toluene tolerant bacterium (Ramos et al. [1995]), while *P. putida* W619 was isolated as an endophyte of poplar (Taghavi et al. [2005]). *Pseudomonas putida* GB-1 was isolated from fresh water and identified as manganese oxidizer (Rosson and Nelson [1982]). None of *P.putida* strain was isolated for PHAs production; however, *P.putida* KT2440 was reported as a PHAs producer (Haywood et al. [1989]; Huisman et al. [1989]).

Polyhydroxyalkanoates (PHAs) are the largest group of biologically produced biodegradable biopolyesters providing different properties and applications (Huijbert and Eggink [1996]; de Smet et al. [1983]). A wide variety of microorganisms accumulate PHAs as a carbon and energy storage material in carbon-excess conditions when a major nutrient (typically nitrogen or phosphorus) is limiting (Elbahloul and Steinbüchel [2009]; Rehm [2010]; Brandl et al. [1988]). Accumulation of excess carbon is a general mechanism used by *Pseudomonas* and is essential for resource balancing (de Eugenio et al. [2010a]; Escapa et al. [2012]). De Smet et al. ([1983]) detected the inclusion bodies in *Pseudomonas olevorans* for first time when grown on octane and identified is as polymer of 3-hydroxyoctanoate. Huisman et al. ([1989]) studied the PHAs synthesis in fluorescent pseudomonads and confirmed that *P.putida, P.fluorescens, P.aeruginosa, P.tetosteroni* and *P. oleovorans* have the capacity to produce PHAs. Among the *P.putida* strains only *P.putida* KT2440, which was isolated as a plant growth promoting bacterium, has been studied for PHAs production.

*Pseudomonas putida* LS46 was isolated from wastewater on the basis of its ability to synthesize novel medium chain-length polyhydroxyalkanoates (mcl-PHAs), which accumulated 22% - 56% of cell dry weight when cultured, under batch culture conditions in media containing glucose, waste vegetable fryer oils or fatty acids (Sharma et al. [2011]). Analysis of *P. putida* LS46 16S rDNA displayed more than 99% nucleotide sequence identity to other *P. putida* strains. Complete genome sequence of other *P.putida* strains which were developed for plant growth promotion or degradation of xenobiotic, are available. Last genome comparison of *P. putida* strain W619 identified genes for aromatic pathways, heavy metal resistance and plant growth promotion in *P.putida* strain KT2440, F1 and GB-1 (Wu et al. [2011]). Since then six new *P. putida* strains, including *P. putida* LS46 genomes have been added to the database. The objective of this work is to see how the genomes of *P.putida* identified as either as biocontrol or bioremediation agents differ from each other due to their geographic origin, environment. Keeping this in mind the genome sequence of *P. putida* LS46 was compared with the genomes of nine other *P. putida* strains for general genome features, shared gene analysis, occurrence of insertion sequences, prophages, genomic islands and for metabolic diversity including PHAs production. The present study revealed more similarity than variability among the genome of *P. putida* strains isolated from different ecological niches in different locations.

## Materials and methods

### Genome sequences and accession numbers

*P putida* LS46 was isolated from a municipal wastewater treatment plant in Winnipeg, Manitoba, Canada, and the strain was deposited with International Depository Authority of Canada (IDAC) at the National Microbiology Laboratory, Health Canada Culture Collection (NML-HCCC), WDCM number 840 (Sharma et al. [2011]). The *P. putida* LS46 genome was sequenced by the Genome Canada sequencing facility at McGill University, Montreal, Quebec, using a combination of Illumina Gaii (Bennett [2002]) and 454 pyro-sequencing technology (Margulies et al. [2005]). The draft genome sequence of *P.putida* LS46 was deposited in the NCBI GenBank database with Accession number ALPV02000000 (Sharma et al. [2013]). The complete genomes of nine *P. putida* strains KT2440 (NC002947), F1 (NC009512), BIRD1 (NC017530), GB-1 (NC010322) S16 (CP003734), ND6 (CP_003589), DOT-T1E (NC_018222), UW4 (CP_003890) and W619 (NC010501) were obtained from the Joint Genome Institute database (http://img.jgi.doe.gov) and compared with the newly sequenced *P. putida* LS46. General genome features like genome size, total number of genes, number of coding sequences (CDS), number of Clusters of Orthologous Groups (COGs) were compared among the genomes of the 10 *P. putida* strains.

### Phylogenetic relationship among *Pseudomonas* strains

The *P. putida* LS46 chaparonin gene (*cpn*60) universal target (UT) sequence was obtained from the *P. putida* LS46 genome sequence and aligned with the *cpn*60 UT sequences of 29 *Pseudomonas* strains available in the Integrated Microbial Genome database Markowitz et al. [2006]). The sequences were aligned using Bioedit (Thompson et al. [1994]) and phylogenetic analysis was conducted using MEGA5 (Tamura et al. [2011]). Phylogenetic tree was constructed using the neighbor-joining method (Saitou and Nei [1987]).

### Occurrence of prophage, insertion sequence and genomic islands

The ten *P. putida* genomes were analyzed for presence of prophages using the PHAST (PHAge Search Tool) prophage finder database (Zhou et al. [2011]). Insertion Sequence (IS) elements in the *P. putida* genomes were identified using the ISfinder tool (Siguier et al. [2006]; Varani et al. [2011]). Genomic islands in *P.putida* genomes were identified using Island finder web based tools (Langille and Brinkman [2009]).

### Whole genome alignments, BLASTn and Pan-genome analysis

Whole genome identity was calculated from pair-wise comparison of genomes using BLASTn analysis. Comparative synteny Dot plot analysis of the *P. putida* strains was carried out and nine plots of comparison between *P. putida* LS46 and nine *P. putida* strains were obtained (Huang and Zhang [2004]). Maps showing *P. putida* strains were prepared using the Gview server *P. putida* LS46 was compared with the other eight *P. putida* strains using Pan genome analysis from Gview server (Petkau et al. [2010]). Shared genes in different strains were calculated using the Phylogenetic profile program using a single gene profile from the Integrated Microbial Genome server. The number of homologous genes in genome 1 in comparison to genome 2 was counted. Reciprocally, the numbers of homologous genes in genome 2 in comparison to genome 1 were counted. Percent shared genes were calculated by adding the homologous genes in genome 1 and 2, dividing by the total number of genes in genome 1 + 2, and multiplied by 100 (Palmer et al. [2012]).

### Comparison of metabolic genes

Genes related with different metabolic functions were identified by searching for homologous genes in *P.putida* genomes by using BLAST analysis of find gene in IMG web page using default parameter (Integrated Microbial Genome). Homologous genes for aromatic compound degradation, heavy metal resistance, manganese oxidation, nicotinic acid degradation, iron scavenging were identified using BLASTn analysis of IMG web page. Likewise TonB dependent receptor and dioxygenase genes were identified in different *P.putida* strains.

## Results

### Phylogenetic relationship of *P. putida* LS46 to other *Pseudomonas* strains

**Figure 1:**
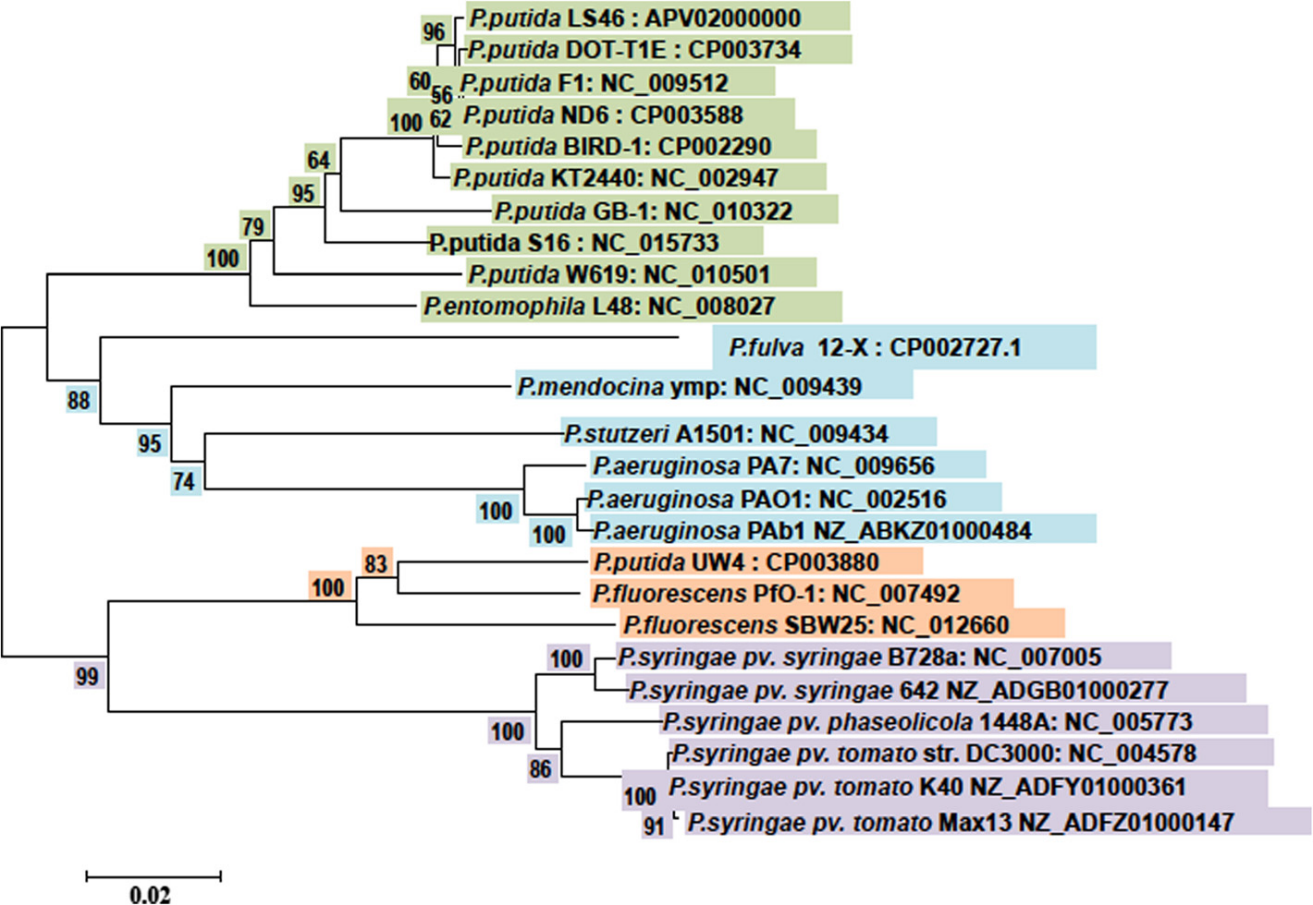
**Phylogenetic tree depicting the relationship among*Pseudomonas*species.** The tree is based on *cpn60* gene sequences, which were aligned by ClustalW and a neighbor-joining tree was generated using MEGA5 program. Bootstrap values are mentioned at the node.

A polyhydroxyalkanoate producing bacterium was isolated from wastewater and was identified as a strain of *Pseudomonas putida* on the basis of 16S rRNA gene sequence. The16S rRNA gene sequence analysis revealed more than 99% nucleotide sequence identity to other *P. putida* strains. Protein-encoding genes are known to provide higher levels of taxonomic resolution than non-protein-encoding genes like 16S rRNA gene. Therefore, a protein coding gene *cpn60* (Hsp60 or GroEL) was used for phylogenetic analysis of *Pseudomonas* species and strains. Neighbor joining trees based on *cpn60* genes divided the *Pseudomonas* species into two clades, one containing *P. putida, P. entomophila, P. mendocina, P. fulva P. aeruginosa* and *P. stutzeri*. The other clade included *P. fluorescens* and *P. syringae* strains. Although *P.putida* strains were clustered in one subclade yet minor differences in *cpn60* gene sequence separated these strains from each other. *Pseudomonas putida* LS46 was closely related to *P. putida* strains ND6, F1, DOT-T1E, BIRD1, KT2440 and clustered with these strains forming a sub-clade (Figure 1). *Pseudomonas putida* strains LS46 was more distantly related to *P.putida* strains GB1, S16 and W619. *Pseudomonas putida* UW4 was not related to any other *P. putida* strains and clustered with *P. fluorescens*.

### Genome features of *P. putida* LS46

The complete genome *P.putida* LS46 was assembled in 32 contigs and consists of 5,874,759 bp DNA with the G + C content of 61.69%. It was predicted to encode 5346 genes, with 5248 CDSs (98.17% of predicted genes). Coding region covers 88.48% of the whole genome. Out of 5248 CDSs, 3889 were assigned functions and 1359 CDSs encoded hypothetical proteins. Among these CDSs, 4270 (79.87%) genes could be classified into COG families composed of 22 categories. A total of seven rRNA operons including seven 5S rRNAs, seven 16S rRNAs, and eight 23S rRNAs were present on the chromosome. In addition, 76 tRNA genes that represent all 20 amino acids, and a tRNA for selenocysteine, were identified (Table 1). No plasmid was detected in *P.putida* LS46. BLASTn analysis identified 181 unique genes (less than 30% minimal identity and maximum e-value of 1 e-^5^) in *P. putida* LS46, which were not present in other nine other *P. putida* strains. Most of the unique genes were predicted to code hypothetical proteins.

**Table 1:** **General features of the genomes of** ***P. putida*** **strains**

**Strain**	**BIRD1**	**DOT-T1E**	**F1**	**GB-1**	**KT2440**	**LS46**	**ND6**	**S16**	**UW4**	**W619**
Isolated in	Spain	Spain	USA	USA	Japan	Canada	China	China	Canada	USA
Genome size	5731541	6260702	5959964	6078430	6181863	5862556	6304310	5984790	6183388	5774330
% G + C	61.74	61.43	61.86	61.94	61.52	61.69	61.62	62.32	60.05%	61.44
Gene count	5046	5802	5423	5515	5481	5316	6484	5307	5517	5292
Homologous genes (%)^a^	92.62	91.39	93.75	90.17	91.32	100	85.57	89.97	82.02	89.14
% Homology^b^	95.89	97.67	97.52	90.86	95.94	100	97.51	91.41	86.59	87.06
CDS count	4960	5721	5300	5417	5350	5219	6391	5218	5423	5194
CDS %	98.30	98.60	97.73	98.22	97.61	98.17	98.57	98.32	98.30	98.15
16S rRNA	7	0	6	7	7	7	6	6	7	7
tRNA	64	58	76	74	74	76	74	70	72	75
COG count	4139	4509	4171	4267	4199	4260	4519	4313	4541	4089
% COG	82.02	77.71	76.91	77.37	76.61	78.61	69.69	81.27	82.31	77.27
Protein W FP^c^	75.23	71.35	73.45	74.75	66.30	79.50	64.24	76.37	75.37	74.79
Protein w/o FP ^d^	23.07	27.25	24.29	23.37	31.31	17.29	34.33	21.95	22.93	23.26
Reference	Matilla et al. [2011]	Udaondo et al. [2012]	Wu et al. [2011]	Wu et al. [2011]	Nelson et al. [2002]	Sharma et al. [2013]	Li et al. [2012]	Yu et al. [2011]	Duan t et al. [2013]	Wu et al. [2011]

^a^ 80% minimal nucleotide sequence identity; e-value 1 e^−5^.

^b^BLASTn of whole genome.

^c^ Proteins with function prediction (%).

^d^ Proteins w/o predicted function (%).

### Insertion Sequence in *P. putida* LS46

Blast analyses of the *P. putida* LS46 genome with IS finder database identified three complete (ISPa41, ISPs1, ISPa42) and one partial (ISPpu9) IS elements (Additional file 1: Table S1). IS element ISPa41, which belongs to IS5 family, was earlier identified in *P. aerugonosa* DK2, *P. putida*, and *P. syringae* DC3000 (IS finder database). Blasting of ISPa41 sequence of *P.putida* LS46 identified homologous sequences in *P.putida* strain GB-1, KT2440 and ND6 but not in *P.putida* BIRD1, DOT-T1E and UW4. Partial sequences of ISPa41 were also present in *P.putida* F1, S16 and W619. ISPs1 was originated from *P. syringae* pv. *syringae* plasmid pPSR1 and *P. syringae* pv. *savastanoi*. ISPs1 from *P. putida* LS46 had 84% nucleotide sequence identity to ISPs1 from *P. syringae* plasmid pSR1. Complete ISPsI was also present in *P.putida* GB-1 and ND6. Incomplete ISPsI was also detected in *P.putida* strain S16, F1 and W619. Complete ISPpu9 from *P. putida* KT2440 was 2043 bp but in *P.putida* LS46 partial ISPpu9 was present (1697bp). It showed 98% sequence identity to ISPpu9 from *P. putida* KT2440. The ISPpu9 integration target site is 23 bp sequence, which is a part of REP identified in *P. putida* KT2440 (Aranda-Olmedo et al. [2002]). ISPpu9 was also present in *P.putida* BIRD1 but was absent in *P.putida* F1, GB1, DOT, ND6 UW4 and W619.

ISPa42, which is synonymous to ISPsy10, encodes a Tn3-like transposable element, which is thought to originate from *P. aeruginosa* DK3. ISPa42, a complete *Tn*3 element of 16941 bp in *P. aeruginosa* DK2, was detected in *P. putida* LS46, but was disrupted by an intergenic region of 19922 bp. It bifurcated it into two segments of 4765 and 12176 bp. This 17 kb transposon was a deletion derivative of Tn4661 toluene degradation transposon Tn4651. The 19.9kb inergenic region of Tn3 carried heavy metal resistance genes like czcA efflux protein (PPUTLS46_008564) *copA* (PPUTLS46_008564), *copB* (PPUTLS46_08569), copper translocating P-type ATPase (PPUTLS46_008644), blue copper domain (PPUTLS46_008654), two component metal regulator sensor signal (PPUTLS46_8669) and heavy metal sensor signal transduction histidine kinase (PPUTLS46_008674) along with sterol desaturase (PPUTLS46_008539). This transposon was unique and was not present in any other *P.putida* strains. Tn4651, a toluene transposon, carried genes for toluene degradation (*xyl*) genes (Tsuda et al. [1989]). *P.putida* LS46 did not have toluene degradation ability due to deletion of toluene genes in Tn4661. A number of catabolic transposons have been identified in *Pseudomonas* species for toluene, naphthalene, chlorobenzoate, chlorobenzene and halogenated alkanoates (Wyndham et al. [1994]). Absence of these catabolic transposons in *P.putida* LS46 limited it metabolic diversity.

### Prediction of prophages

Prophage finder (PHAST) identified one intact prophage and one putative phage genes in *P. putida* LS46 genome. The intact prophage sequence was was present on Contig ALPV02000012 (18373 to 68052) and was 49.6 kb in size with G + C ratio of 59.64%. A total of 61 CDSs which included 40 phage proteins, 21 hypothetical proteins and one tRNA were identified in this prophage. *P.putida* LS46 prophage showed upto to 73% nucleotide sequence identity to *Pseudomonas aeruginosa* phage *Pseudomonas* vB_PaeS_PMG1 which is a virulent phage with lytic infection cycle (Krylov et al. [2012]). A 79 bp attachment sites (attL and attR) were also present. This phage was also present in *P.putida* BIRD1, *P.putida* KT2440 and *P.putida* W619 genomes (Additional file 1: Table S2). However the possible prophages are decided on the basis of number of CDS shared with other prophages. *P.putida* LS46 shared 15 CDS with *Pseudomonas* vB_PaeS_PMG1 while *P.putida* BIRD1, *P.putida* KT2440 and *P.putida* W619 had 8, 14 and 15 CDS respectively. The organization of prophage *Pseudomonas* vB_PaeS_PMG1 genes was unique to *P.putida* LS46. The putative prophage was only 8.4 kb and had only 9 protein coding sequences. It was similar to SXt2_1717. Other *P. putida* genomes (from PHAST database http://phast.wishartlab.com) were predicted to contain 1–6 (intact, incomplete, and questionable) prophages (Additional file 1: Table S2). No prophage was detected in *P. putida* UW4.

### Genomic islands in *P.putida* LS46

**Figure 2:**
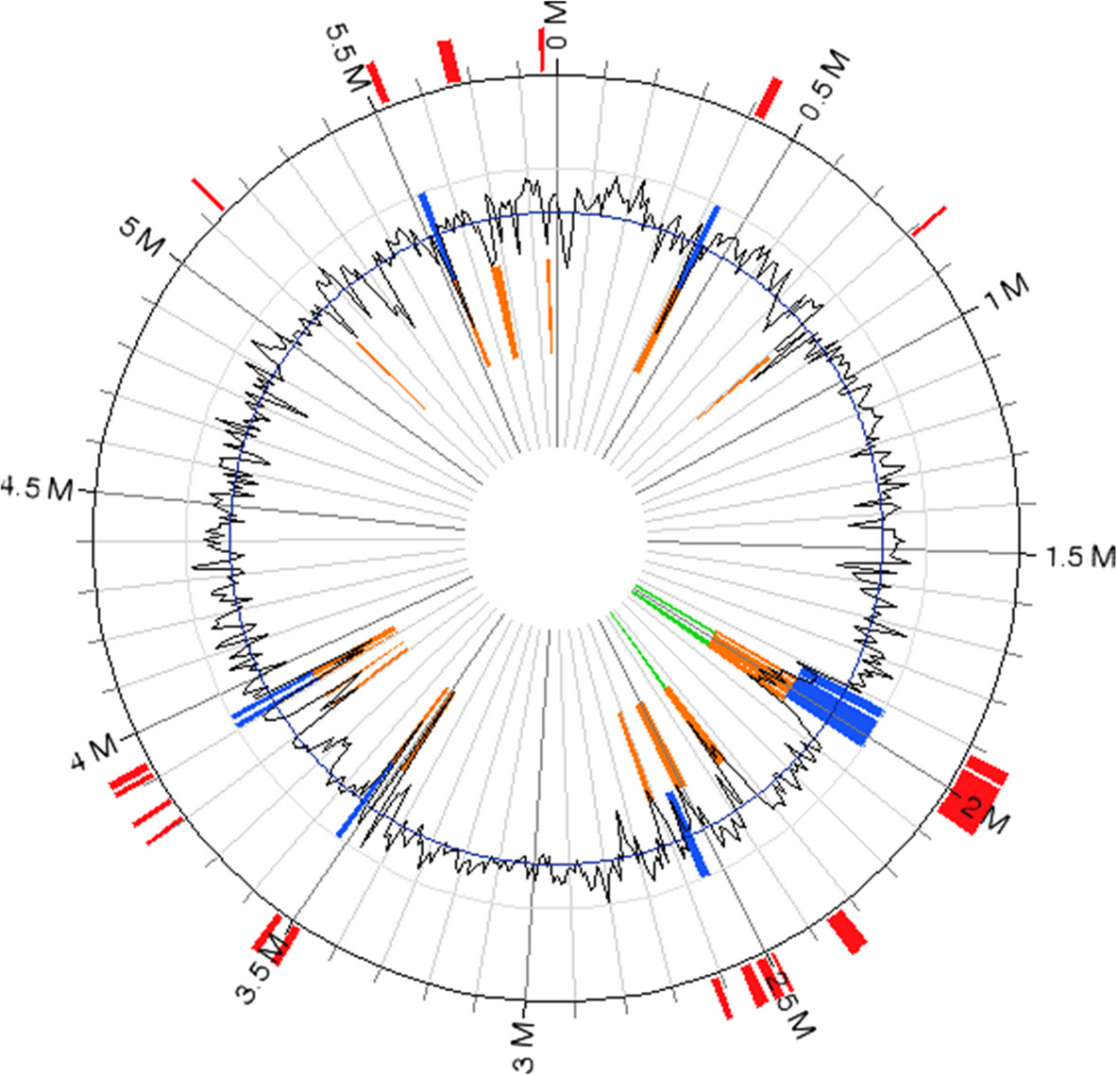
**Occurrence of genomic islands in*P.putida*LS46 genomes.** Genomic islands were identified using IslandViewer programme using Genomic island predictions were calculated for using Integrated method of IslandPick, IslandPath-DIMOB, and SIGI-HMM (Langille and Brinkman ([2009])).

Genomic island finder using integrated method identified 22 genomic islands (GIs) in *P.putida* LS46 (Figure 2). The size of smallest genomic island was 4505 bp while largest was 78290 bp (Figure 2, Additional file 1: Table S3). Genomic island 3 was 26639 bp in size and it showed 96% homology to *P.putida* H8234 genome (Molina et al. [2013]). This GI carried a transposon Tn4652 along with heavy metal resistance genes. *P. putida* H8234, a clinical isolate from France, showed low pathogenic potential compared with *P. aeruginosa* and was resistant to commonly used antibiotics. *P.putida* LS46 was also resistant to ampicillin, choloramphenicol, gentamycin and tetracycline. GI 18 contains M.XmaI and R.XmaI genes, which are component of XmaI restriction system. Two unique genes PPUTLS46_017749, which encodes an N-4 cytosine-specific methyltransferase, and PPUTLS46_017754, which encode a Type II restriction enzyme, encoded this system. This restriction system is isoschizomer of *XcyI* of *Xanthomonas campestris* pv *cynopsidis.* The *XcyI* restriction-modification system recognizes the sequence, CCCGGG, and cleaves after C1 and (Withers et al. [1992]). These genes are present on the plasmid AG1 of *Xanthomonas axonopodis* pv *glycine* as well as on the plasmid pRA2 of *Pseudomonsa alcaligenes* NCIB 9867. This plasmid carried two mobile elements, Tn5563 and IS1633 along with Pac25I (XcyI) restriction-modification system (Kwong et al. [2000]). This restriction system did not affect the pRA2 plasmid stability in heterologous *Pseudomonas* hosts. The other GIs carried a number hypothetical protein.

### Comparative genome analysis of *P.putida* strains

The genomes of nine *P. putida* strains range in size from 5.73 to 6.3 Mb, with an average GC content of 62% (Table 1). Among *P. putida* strains only strain ND6 carried two plasmids of 117 kb and 101 kb, which encoded an additional 136 and 102 genes, respectively (Table 1). *P. putida* strains KT2440, BIRD1, GB1, and LS46 had seven copies of 16S rRNA genes, while six copies of 16S rRNA genes were present in strains F1, ND6, UW4, and S16 (Table 1). *P. putida* strains carried 4171 to 4519 COG counts, representing 69.7% to 82.0% of their genes.

Genome of nine *P.putida* strains including *P.putida* LS46 was blasted against *P.putida* KT2440 (type strain) as the reference strain. Whole genome BLAST identified several homologous regions as well as diverse region in the *P. putida* strains in comparison *to P.putida* KT2440. On the basis of whole genome BLASTn analysis of nine *P. putida* genomes had 86.6% to 97.7% nucleotide sequence identity to the *P. putida* LS46 genome (Table 2). *P. putida* DOT-T1E displayed the greatest sequence identity with *P. putida* LS46, while *P. putida* UW4 had the least sequence identity. *P.putida* DOT-T1E was isolated from Spain after enrichment with 1% toluene while *P.putida* UW4 was isolated as PGPR from Canada (Ramos et al. [1995]; Glick [1995]).

**Table 2:** **Percent shared genes among different** ***Pseudomonas putida*** **strains**

**Strain**	**LS46**	**BIRD1**	**DOT-T1E**	**F1**	**GB1**	**KT2440**	**ND6**	**S16**	**UW4**	**W619**
LS46	100									
BIRD	92.54	100								
DOT-T1E	91.60	91.90	100							
F1	93.67	92.72	93.99	100						
GB1	90.13	89.36	92.16	89.83	100					
KT2440	91.28	89.38	89.96	90.32	87.90	100				
ND6	85.67	85.13	86.96	86.90	83.13	84.31	100			
S16	89.85	89.15	88.14	90.55	90.12	89.11	83.86	100		
UW4	82.05	82.54	79.64	81.77	81.82	81.91	75.32	82.20	100	
W619	89.07	88.78	88.22	89.69	87.74	87.42	83.29	89.52	81.84	100

### Genome arrangement of *P. putida* genomes

**Figure 3:**
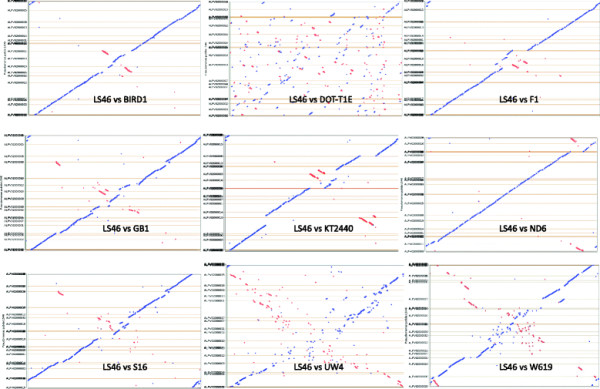
**Comparative Synteny Dot plot of*P.putida*strains showing orthologous relationship of*P. putida*LS46 with nine*P.putida*strains.** The analysis was carried out using the Dot plot from Integrated Microbial Genome (IMG) website.

Genome arrangement of *P.putida* LS46 was compared with 9 other *P.putida* strains using Dot plot analysis (Figure 3). Dot plot analysis of *P.putida* LS46 with other *P.putida* genomes indicated the similarities in genome arrangement at the nucleotide level. The diagonal line showed the co-linearity DNA strands. The blue block on the left hand indicated the translocation and inversions in the genomes. The red blocks represented translocations in anti parallel strands of the genome. The high degrees of genome similarity as well as differences in the arrangement were apparent among *P.putida* genomes. The genome wide distribution of conserved region of *P. putida* strains varied from strain to strain. Organization of the *P. putida* LS46 genome was clearly different than other strains and a number of inversions and translocations were observed the genome in comparison to other *P.putida* genomes (Figure 3). On the basis of genome arrangement *P.putida* strains could be divided into two groups. In first group *P.putida* strains LS46, ND6, F1, KT2440, GB-1, S16 and BIRD1 can be placed which had significantly similarity to *P.putida* LS46 genome arrangement while in second group comprises of *P.putida* W619, DOT-T1E and UW4 which had low similarity to *P.putida* LS46. *P.putida* LS46 genome arrangement was markedly different from *P.putida* DOT-T1E, W619 and UW4 with large number translocations and inversions. *Pseudomonas putida* LS46 genome showed large conserved blocks that are also present in *P. putida* BIRD1, F1, and ND6 strains, while *P. putida* DOT-T1E had a large number of small conserved blocks. In comparison to *P. putida* LS46, the genomes of *P.putida* KT2440 and S16 genomes had more inversions.

### Homologous and shared gene among *P.putida* strains

**Figure 4:**
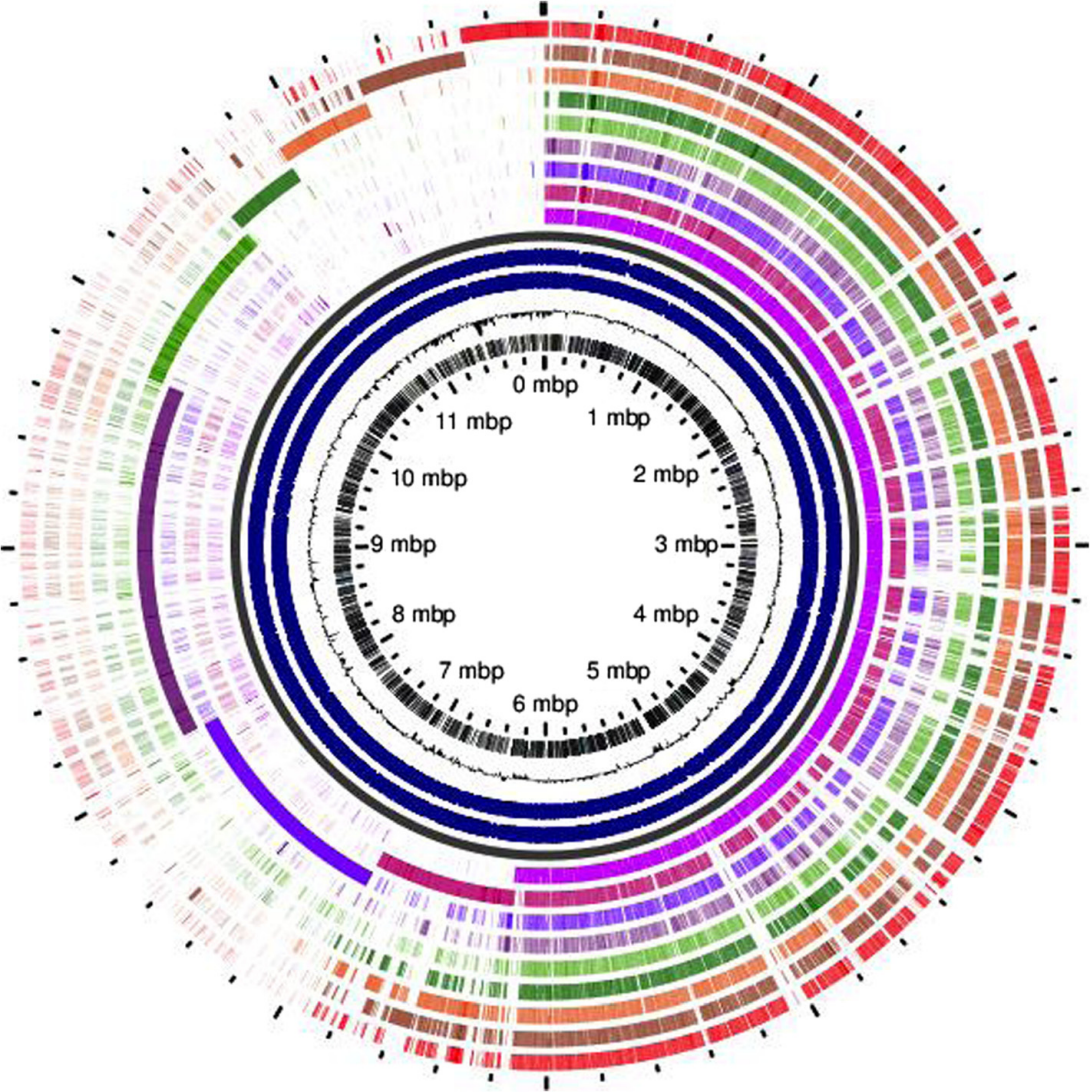
**Pangenome analysis of nine*P. putida*strains with*P. putida*KT2440 as a reference.** From inside to outside the circles. COG categories, GC content, backbone, COG in positive strand of pangenome, COG in negative strand in pangenome, *P. putida* KT2440, *P. putida* F1, *P. putida* GB-1, *P. putida* W619, *P. putida* S16, *P. putida* BIRD-1, *P. putida* ND6, *P. putida* DOT-T1E and *P. putida* LS46.

BLASTn analysis of all genes of *P. putida* LS46 (80% minimal identity e value 1^−5^) against nine genome identified 82.02- 93.75% homologous genes encoded by the *P. putida* strains were shared by the ten genomes. *P.putida* F1 shared highest number (93.7%) of genes with *P.putida* LS46 while *P.putida* ND6 (85%) and *P.putida* UW4 (82%) least number of genes with *P.putida* LS46 (Table 1). Using single gene profiler 3271 genes were identified which were present in present in all *P.putida* strains. A total of 8786 core and unique genes were represented the pangenome of nine *P. putida* strains (excluding *P.putida* UW4). Unique region in *P. putida* genomes were identified using pangenome analysis, which identified unique genes present in only one strain (Figure 4). However, all genomes showed higher functional identity (presence of COGs) reflected by high correlation coefficients among the different genomes (r^2^ = 0.94), although the distribution of different COGs categories among the different genomes was different and represented the functional diversity. The number and percentage of different COG categories varied greatly among ten *P. putida* strains. *P.putida* LS46 had highest number of COGs with unknown function. *P.putida* LS6 genome arrangement was strikingly different from *P.putida* DOT-T1E however both the strains shared higher percentage of genes (91.6%). Inversely *P.putida* LS46 and *P.putida* ND6 had significantly similar genome arrangement but % of shared genes between two genomes was low (85%).

### House keeping genes

The 16S rDNA is an universal marker and has been widely used for comparison between divergent bacteria. However, the resolution of 16S rRNA gene sequences at the intrageneric level is low Anzai et al. [2000]; Yamamoto et al. [2000]). Gene sequences for ‘housekeeping’ proteins provide better phylogenetic resolution and have been used to differentiate the genomes of closely related strains. Zeigler ([2005]) identified some house keeping genes for studying genome relatedness among different strains. Comparison of 33 house keeping genes of *P. putida* LS46 with other nine *P.putida* strains revealed a high degree of homology among these genes (Additional file 1: Table S4). However, some house keeping genes like *dnaX* (PPUTLS46_019951), *lig* (PPUTLS46_022046), *pgi* (PPUTLS46_007236), *uvrC* (PPUTLS46_012340,) *glyA* (PPUTLS46_024688), *trpS* (PPUTLS46_013403) and *trmE* (PPUTLS46_016619) were highly diverse among *P. putida* strains (Additional file 1: Table S4).

### Fatty acid and Polyhydroxyalkanoate synthesis

Polyhydroxyalkanoates are produced *Pseudomonas putida* LS46 from glucose, glycerol, biodiesel glycerol, fatty acids and waste fryer oil (Sharma et al. [2011]). Major components of PHAs produced by *P.putida* LS46 are either 3-hydroxyoctonoate or 3-hydroxydecanoate depending on the carbon substrate used for PHAs production (Sharma et al. [2011]). There are six genes in the polyhydroxyalkanoate (PHA) synthesis operon (*pha*) in *P.putida*. These are *phaC1, phaZ, phaC2, phaD, phaF* and *phaI.* The *phaC1* and *phaC2* are PHA synthases (type II) that incorporate (R)-3-hydroxyacyl-CoA monomers into the PHA polymer (Huisman et al., [1989]). The *phaZ* encodes a PHA depolymerase, which hydrolyses the PHA monomers, which can be fed into central metabolism for growth (Galán et al. [2011]; de Eugenio et al., [2010a]). The other three genes (*phaD, phaF* and *phaI*) are regulatory genes (Arias et al. [2013]; de Eugenio et al. [2010b]; Galán et al. [2011]). The organization of *pha* operon was identical in all the *P. putida* strains. The PHA synthesis gene products were highly conserved among all the *P. putida* strains, with more than 90% amino acid sequence identity (Table 3). The two-polyhydroxyalkanoate synthase genes (*phaC1* and *phaC2*) had only 71% nucleotide identity and 55% aa sequence identity.

**Table 3:** Homology of proteins associated with polyhydroxyalkanoate production

**Name**	**Locus tag**	**Enzyme**	**Product size (aa)**	***P.putida*** **strains**							
				**BIRD1**	**DOT**	**F1**	**GB1**	**KT2440**	**ND6**	**S16**	**W619**
FadD	07414	Acyl-CoA synthetase	565	99	100	99	94	99	99	94	87
FadA	04404	3-ketoacyl-CoA thiolase	392	99	100	100	99	99	100	99	100
FadAx	07424	Acetyl-CoA acetyltransferases	380	99	99	99	98	99	99	98	96
FadB	00695	3-hydroxyacyl-CoA dehydrogenase/epimerase	715	98	93	99	94	94	99	93	95
FadB2	07419	3-hydroxyacyl-CoA dehydrogenase	255	99	100	100	97	100	99	94	96
FadD2	21046	Acyl-CoA synthetase	562	99	99	99	98	99	99	96	93
FadE	00145	Acyl CoA dehydrogenase	601	98	93	99	94	94	99	93	95
FadE2	05901	Acyl CoA dehydrogenase	592	95	99	99	95	98	99	95	94
PhaC1	05621	Poly(R)-hydroxyalkanoic acid synthase	560	99	99	98	99	99	99	98	94
PhaZ	05616	Poly(3-hydroxyalkanoate) depolymerase	283	99	100	100	99	99	99	97	95
PhaC2	05611	Poly(R)-hydroxyalkanoic acid synthase	559	99	99	99	96	99	100	99	95
PhaD	05606	Transcriptional regulator	204	100	99	96	99	99	99	97	95
PhaF	05601	PHA granule associated protein	253	99	100	100	97	99	100	98	94
PhaI	05596	PHA granule associated protein	139	99	100	96	93	96	96	91	91
PhaG	13888	Hydroxyacyl-ACP:CoA transacylase	295	99	99	99	98	99	100	94	89
FabD	00250	malonyl CoA-acyl carrier protein transacylase	312	99	99	100	96	99	100	98	92
FabA	12710	3 hydroxyacyl(decanyl)-ACP dehydratase	171	100	100	100	99	100	100	98	92
FabB	12715	2 -Oxoacyl carrier protein synthase	406	100	99	99	99	99	99	99	99
FabG	23353	3-ketoacyl-(acyl-carrier-protein) reductase	450	99	99	99	96	99	99	95	90
FabG_2	00255	3-oxoacyl-(acyl-carrier-protein) reductase	450	99	100	99	99	100	99	100	99
FabG_3	15924	3-oxoacyl-(acyl-carrier-protein) reductase	450	100	100	100	97	99	99	94	91
FabZ	14589	3-hydroxyacyl-[acyl carrier protein] dehydratase	146	100	100	100	100	100	100	99	95

**Figure 5:**
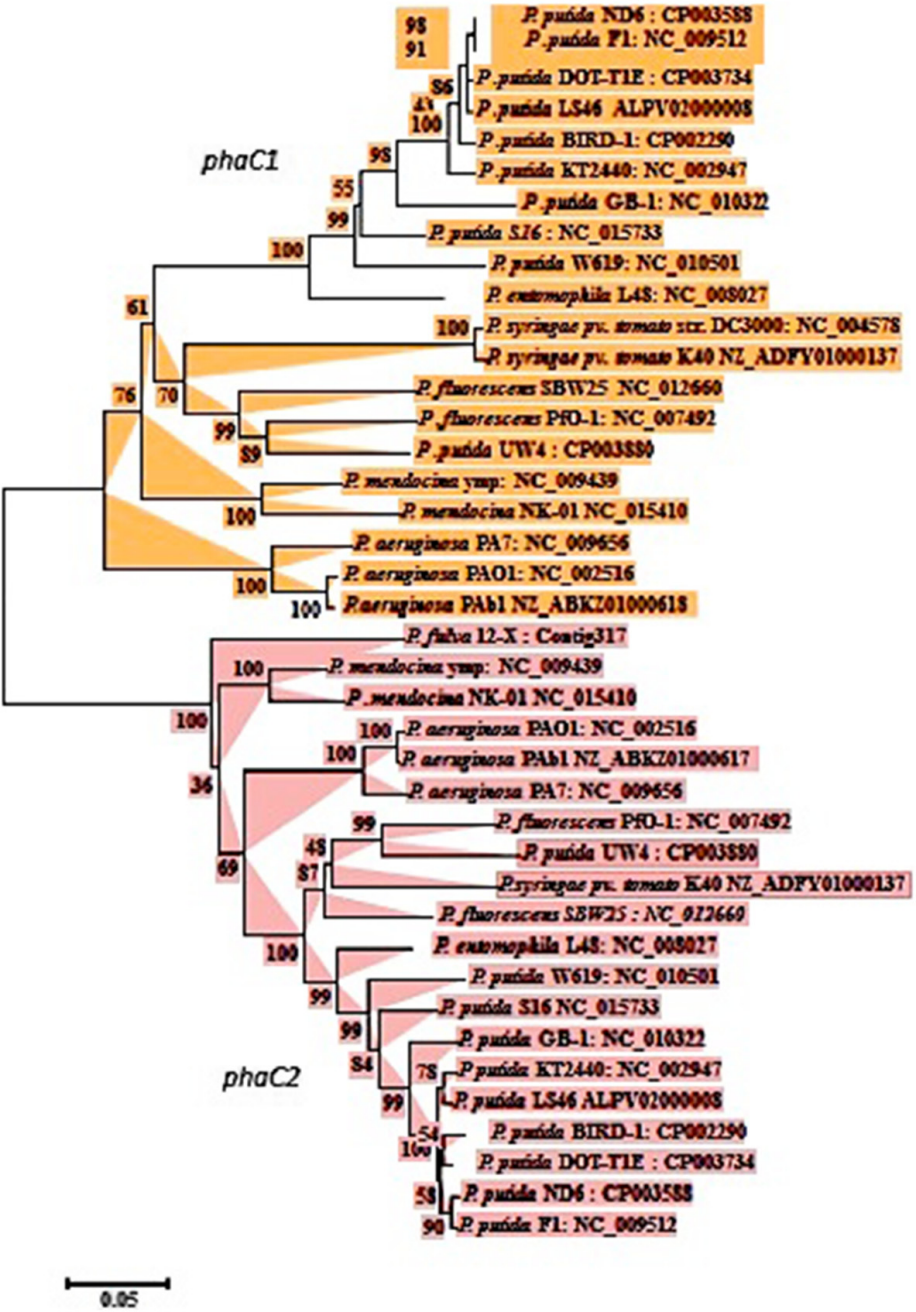
**Phylogenetic tree depicting the relationship of*phaC1*and*phaC2*genes among*Pseudomonas*species.** The *phaC* gene sequences were aligned by ClustalW and a neighbor-joining tree was generated using MEGA5 program. Bootstrap values are mentioned at the node.

The *phaC1* and *phaC2* of different *Pseudomonas* species formed different clusters in neighbor joining tree. The *phaC1* and *phaC2* in *P.putida* strains were highly conserved but were different *phaC1* and *phaC2* from other *Pseudomonas* species. The *phaC1* and *phaC2* genes of *P. putida, P.aeruginosa, P.fluorescens, P.stutzeri. P.entomophila and P.mendocina* formed different cluster in neighbor joining tree (Figure 5)*.* Further fatty acid biosynthesis and fatty acid degradation proteins were highly conserved among *P.putida* strains. Fatty acid biosynthesis (*fab*) and fatty acid degradation (*fad*) gene products provide the precursor for PHAs synthesis. Most of the fatty acid synthesis and degradation proteins of *P.putida* LS46 had multiple genes coding isozymes i.e. FadB had 4 isologs, FadA had 5 isologs and FadD had 7 isologs for short and long chain fatty acids. Likewise FadE (acyl-CoA dehydrogenase) had six isologs specific for small, medium and long chain fatty acids. Fab and Fad proteins of *P.putida* LS46 showed high homology to Fab and Fad proteins of other *P.putida* strains. *P.putida* LS46 can utilize fatty acids (C5-C18) for PHAs production. Two fatty acid transporters (FadL), one for short chain fatty acid (PPUTLS46_007654) and other for long fatty acid (PPUTLS46_015009) were present in *P.putida* LS46. However, it preferentially used long chain fatty acids (C6-C18) than short chain fatty acid C3-C5) for PHAs synthesis. The specificity of FadD and FadL these proteins are not known but transfer of FadD from *E.coli* and FadL from *P.putida* into *Aeromonas hydrophila* improved its ability to utilize C6 and C8 fatty acids. (Jian et al. [2010]). The intermediate 3hydroxyacyl-ACP of *de novo* fatty acid synthesis is converted to 3hydroxyacyl-CoA for polymerization to PHAs with help of (R)-3-hydroxyacyl-ACP:CoA transacylase enzyme PhaG. PhaG was present in all *P.putida* strains. The absence of PhaG in PHB producers limits their ability to produce mcl-PHAs. Transfer and expression of *phaG* of *P.putida* in *Ralstonia eutropha* or *Aeromonas hydrophila* producer may confer mcl-PHAs production ability into PHB producers.Recently, a PHAs granule-associated acyl-CoA-synthetase (Acs1) has been identified which is highly conserved among P.putida strains. It directs the carbon flux of these central metabolites towards PHA accumulation and converts 3hydroxyalkanoic acids to 3 hydroxyacyl-CoA thioesters (Ruth et al. [2008]).

### Comparison of common metabolic pathways of *P. putida* strains

#### Metabolic diversity

The compared *P. putida* strains were isolated from diverse environment for bioremediation of xenobiotics or decomposition of other materials. Different *P. putida* strains have unique genes associated with specific functions and these genes are not present in other strains. *P. putida* KT2440 has been considered as a metabolically diverse saprophytic bacterium. Analysis of the *P. putida* KT2440 genome identified 18 dioxygenase, 51 putative hydrolases, 40 dehydrogenases, and more than 62 transferases (Jimenez et al. [2002]; Molina-Henares et al. [2009]; Nelson et al. [2002]). By way of comparison, the *P. putida* LS46 genome had 22 dioxygenases (Additional file 1: Table S5), 75 hydrolases, 149 dehydrogenases, and 43 transferases. All the three dioxygenases, benzoate dioxygenase (PPUTLS46_007854, PPUTLS46_007859), catechol dioxygenase PPUTLS46_007879), and protochatuate dioxygenase (PPUTLS46_010694) were present in *P. putida* LS46. These dioxygenases were earlier identified in *P.putida* KT2440 and are involved in metabolism of aromatic compounds (Nelson et al. [2002]). *P.putida* LS46 had 22 dioxygenases genes. One of the dioxygenase genes (PPUTLS46_007879 glyoxalase/bleomycin resistance protein/dioxygenase) was unique to *P.putida* LS46 and was not present in other *P.putida* strains (Additional file 1: Table S5).

The majority of the genes for degradation of ferulate, coniferyl, and coumuryl alcohol, aldehydes and acids, p-hydroxybenzoate, and protocatechuate present in *P. putida* KT2440 were also present in *P. putida* LS46 (Additional file 1: Table S6). These genes were reported to have role in degradation of aromatic/aliphatic sulphonate, benzoate, toulate, catechol, hippurate, maleate, phenylalanine, phenylacetic acidprotocatechuate, quinate, taurine etc. The notable exceptions in *P. putida* LS46, however, were ferulic acid transferase (PP_3354), transferuloyl CoA hydratase (PP_3358), and vanillate dehydrogenase (PP_3357). These genes were present in *P. putida* F1 (Pput_2404, Pput_2400, Pput_2401) and *P. putida* W619 (PputW619_2051, PputW619_2047, PputW619_2048), and are associated with transformations of ferulic acid to vanillin and vanillin to protocatechuate. Another gene, which was only reported in *P. putida* KT2440, and that is missing from all other *P. putida* strains, was propanediol dehydrogenase (PP_2803).

*P. putida* F1 is another metabolically diverse strain that has been demonstrated to oxidize toluene, 3-hydroxyphenyl propionate, and cymene (Finette and Gibson [1988]; Zylstra et al. [1988]). The genes that encode the enzymes for these reactions were also present in *P. putida* DOT-T1E, which encodes the genes (TIE_4277-T1E-4240) for degradation of toluene, 3- hydroxyphenylpropoinate, and cymene (Additional file 1: Table S8), but absent in *P. putida* LS46 and 7 other *P. putida* strains. As reported earlier the genes for degradation of 3 hydroxyphenylpropionate were also present in *P.putida* W619 (Wu et al. [2011]).

*P. putida* KT2440 can use aromatic or aliphatic sulphonates as sulphur sources. The genes for degradation of aromatic or aliphatic sulphonates are encoded in the *ssuFBCDEA* operon (PP_0241-PP_0235) in *P. putida* KT2440. This operon was present in *P. putida* LS46 (PPUTLS46_024748, PPUTLS4_025163, 025168, 025173, 025178, 025183) and all other *P. putida* strains (Additional file 1: Table S6). Three chlorohyrolases i.e. atrazine chlorohydrolase (PP5036, PP2584) and hydroxydechloroatrazine ethylaminohydrolase (PP3209) detected in *P. putida* KT2440 were also identified in *P. putida* LS46 (PPUTLS46_10994, PPUTLS46_005456, and PPUTLS46_008014). However, chloride channel protein of *P. putida* KT2440 (PP3959) was absent in *P. putida* LS46. Pak et al. ([2000]) identified xenobiotic reductase from *P. fluorescens* for the transformation of 2, 4, 6-trinitritoluene (TNT). Its homologue was identified in *P. putida* KT2440 (PP_0920) (Nelson et al. [2002]) and *P. putida* LS46 (PPUTLS46_022986). These genes was also present in other eight *P. putida* strains, but was absent from *P. putida* UW4.

*Pseudomonas putida* DOT-T1E is a solvent-tolerant strain able to grow in the presence of > 1% (v/v) toluene in the culture medium. Its multidrug efflux pumps have been found to play a major role in toluene-tolerance (Rojas et al. [2001]). This ability is mainly conferred by an efflux pump encoded in a self-transmissible 133 kb plasmid named pGRT1 (Molina et al. [2011]). No plasmid was detected in *P. putida* LS46, but the genes encoding an efflux pump (*sepA, sepB, sepC*), were present in the *P. putida* LS46 (PPUTLS46_0133778, 013773, 013768) genome, as reported earlier for *P. putida* strains KT2440, GB1 and W619 (Wu et al. [2011]).

#### Nicotinic acid and nicotine degradation

Nicotinic acid (NA) is a carboxylic derivative of pyridine that is widely distributed in nature as part of pyridine cofactors (NAD and NADP) and alkaloids (e.g., nicotine and anabasine), and it is essential (vitamin B3) for those organisms that are not able to carry out its synthesis. In bacteria and fungi, NA is used as a carbon source. The biochemical pathways involved in the degradation of NA was elucidated (Kaiser et al. [1996]). Jiménez et al. ([2008]) identified a *nic* gene cluster (PP_3939 - PP_3948) in *P. putida* KT2440 responsible for aerobic nicotine degradation. These genes were also present in *P. putida* strain F1, GB1, and W619 as reported earlier Jiménez et al. ([2008]). All the genes of *nic* operon were also present in *P. putida* LS46, BIRD1, ND6, DOT-T1E, and these genes were homologous to genes (89.6% to 100% nucleotide identity) from *P. putida* KT2440 (Additional file 1: Table S7).

Recently, the genome sequence of another nictotine degrading *P. putida*, strain S16, was released (Tang et al. [2012]). *P. putida* S16 degrades nicotine through pyrrolidine pathway. A gene cluster containing six genes encoding PPS_0380 HSP hydroxylase (*hspB*), PPS_4060 maleate isomerase (*iso*), PPS_4059 NFM deformylase (*nfo*), PPS_4058 DHP dioxygenase (*hpo*), PPS_4057 maleamate amidase (*ami*), and PPS_4061 6-hydroxynicotinate 3-monooxygenase (*hna*) was identified in *P. putida* S16 and this gene cluster is designated as *nic*2 cluster. This gene cluster was present on a large genomic island. Both nicotine and nicotinic acid produced 2,5- dihydroxypyridine (2,5-DHP) as an intermediate, which is converted to N-formylmalaemic acid. The N-formylmalaemic acid is degraded to maleic acid and then to fumaric acid as in nicotinic degradation pathway (Wang et al. [2007]). BLAST analysis indicated that these genes were also present in *P. putida* LS46 (PPUTLS46_011665 (*ami*), 11670 (*iso*), 11675 (*nfo*), 11685 (*hpo*) and showed 36.63, 69.35, 55.64 43.73% homology to respective proteins of *P.putida* S16. Other *P.putida* strains showed low homology to the *P. putida* S16 *nic*2 cluster (36.6-65-3%). No homologue of PPS_0380 HSP hydroxylase (*hspB*) was present in *P.putida* LS46 (Additional file 1: Table S8). The *nic* cluster genes of *P. putida* KT2440 showed high nucleotide sequence identity (>95%) to corresponding genes of *P.putida* LS46, as reported earlier in *P. putida* strain W619, F1, and GB1 (Molina et al. [2011]) [59]. *P. putida* KT2440, however, is not able to degrade nicotine. The *nic* cluster of *P. putida* S16 had a GC content of only 48% compared with the 62% GC content of the rest of the *P. putida* genome sequence. The *nic* gene cluster for the nicotinic acid degradation pathway of *P. putida* KT2440 was also detected in *P.putida* BIRD1, *P. putida* ND6, and *P. putida* DOT-T1E, but was absent in the genome of *P.putida* strain UW4.

#### Heavy metal resistance

The level of heavy metal tolerance is very high in *P. putida* W619 in comparison *to* other *P. putida* strains (Canovas et al. [2003]; Taghavi et al. [2009]). In *P. putida* W619, copper, cobalt, zinc, cadmium, arsenate, mercury, nickel chromate, and molybdenum resistance genes are present in two genomic regions (1 and 31). Heavy metal tolerance of *P. putida* LS46 has not been investigated, but this strain encodes genes for heavy metal resistance present in genomic region 1 of *P. putida* W619. These genes are involved in copper, cobalt, zinc, and cadmium resistance. However, the genes located on genomic region 31 of *P. putida* W619, involved in copper and chromate resistance, were absent in the majority of *P. putida* strains, including *P. putida* LS46 (Additional file 1: Table S8). All *P. putida* strains lacked the genes present in genomic region 18 of *P. putida* W619, which were associated with mercury and nickel resistance.

#### Manganese oxidation

*P. putida* GB1 is a Mn (II) oxidizing bacterium (Buzzo [2011]) and attempts have been made to identify the genes related with Mn (II) oxidation by transposon mutagenesis. However, the role of different genes in Mn (II) oxidation is not clear (Caspi et al. [1998]; Brouwers et al. [1999]). Recently, using transposon mutagenesis, Geszvain et al. ([2013]) and Geszvain and Tebo ([2009]) identified two genes, which are homologous to multi-copper oxidases (PputGB1_2447 and PputGB1_2665) in *P. putida* GB1. The genes encoding multi-copper oxidase homologues of P.putida GB1 were also present in *P. putida* LS46 (PPUTLS46_002532 and PPUTLS46_006964), as well as in *P. putida* strains KT2440, F1, ND6, W619, S16, UW4, and DOT-T1E. Only one multi-copper oxidase gene was identified in *P. putida* BIRD1 (Additional file 1: Table S8).

#### Iron scavenging genes in P. putida strains

It is well known that iron deficiency in bacteria limits growth Andrew et al. ([2003]). *Pseudomonas* produces low molecular weight iron-chelating compounds termed ferri-siderophores to scavenge iron (Wiener [2005]). Ferri-siderophores are taken up via outer membrane receptors, which function as gated porin channels (Koebnik [2005]; Ratledge and Dover [2000]). After binding of the ferri-siderophores, transport is mediated by a complex of inner membrane-anchored proteins TonB (Wiener [2005]). Fluorescent pseudomonads respond to iron-deficiency by secreting the yellow-green fluorescent peptidic siderophores called pyoverdines (Meyer [2000]; Cornelis et al. [2009]). Pyoverdines have a conserved chromophore with a variable peptide chain, and each *Pseudomonas* species produces a different pyoverdine (Ravel and Cornelis [2003]; Visca et al. [2007]). Pyoverdine is synthesized by non-ribosomal peptide synthetase (NRPS). Like other *P. putida* strains, genes for pyoverdine synthesis were also present in *P. putida* LS46 (PPUTLS46_025659 and PPUTLS46_012775). The *pvdE* gene (PPUTLS46_012775) encoded a PVD ABC transporter, as in other *P. putida* strains. *P. putida* LS46, like the other *P.putida* strains did not carry the genes for pseudomonine production, which was reported for *P. entomophila* L48 (Vodovar et al. [2006]).

The number and type of TonB siderophore receptors show their diversity to survive in the different environment (Cornelis and Bodilis [2009]). TonB-dependent receptor genes are not constitutively expressed and are regulated by the iron availability (Koebnik et al. [2000]; Bodilis et al. [2009]) and their expression provides additional mechanisms to survive under different niches. Twenty-six (26) TonB siderophore receptor genes were present in the *P. putida* LS46 genome (Additional file 1: Table S9). Across all of the *P. putida* strains, 19–53 TonB genes are present. Comparison of TonB genes (at 80% nucleotide sequence identity) of *P. putida* LS46 identified 26, 28, 26 20, 24, 26, 19, 1 and 15 homologous TonB genes in *P. putida* LS46 strain BIRD1, DOT-T1E, F1, GB1, KT2440, ND6, S16, UW4 and W619, respectively. *P.putida* strain UW4 had 11 TonB genes, which showed low homology (50% nuclotide sequence identity) to other TonB genes of *P. putida* strains. The greatest number of TonB genes (55) was present in *P. putida* strain GB1.

## Discussion

*Pseudomonas putida* strains have been identified as root colonizing bacterium and developed as biocontrol agent. Other *P.putida* strains due to their diverse metabolic potential have been developed as bioremediation agents. Potential of *P.putida* as polyhydroxyalkanoates (PHAs) producer was identified in 1989 (Huisman et al. [1989]). However, PHAs producing *P.putida* strain KT2440 was originally isolated as a root colonizing bacterium (Nelson et al. [2002]). A number of *P.putida* strains were isolated and developed as bioremediation agents but no other *P.putida* strain was isolated for PHAs production. *P.putida* LS46 was isolated and screened for PHAs production and it was proved as a good PHAs producer as *P.putida* KT2440. Complete genome of this bacterium was sequenced to know the similarities and differences among biocontrol, bioremediation and PHAs producing *P.putida* strains.

*P. putida* LS46 strain showed more than 99% identity to 16S rRNA gene of *P.putida* strains irrespective of their geographic origin or their application. *P.putida* classifications based on protein-encoding genes like *gyrB, rec, rpoB, recN,* and *cpn60* alongwith 16S rRNA gene have earlier been proposed (Adékambi et al. [2009]; Arahal et al. [2008]; Zeigler [2005]; Mulet et al. [2010,2013]). Cpn60 (Hsp60 or GroEL), a highly conserved protein found in bacteria, has been widely used for phylogeny, microbial identification, microbial ecology and evolution (Hill et al. [2002,2004]). The phylogeny of *P. putida* strains based on *cpn60* confirmed the close relationship among *P. putida* strains, strengthening the 16S rRNA gene based phylogeny. However, the *cpn60* gene phylogeny was more robust and separated strains that were clustered together in the 16S rRNA gene phylogeny. The *cpn60* (UT) analysis differentiated *P. putida* W619, *P. putida* S16, and *P. putida* UW4 from other *P. putida* strains. *P. putida* W619 was isolated as an endophyte of Poplar, *P. putida* UW4 was isolated as a plant growth promoting species, and *P. putida* S16 was isolated as a nicotine degrading species. Recently *cpn60* (UT) was used for studying the phylogenetic relationship among *Aeromonas, Themoanaerobacter*, and vaginal microbiota (Miñana-Galbis et al. [2009], Verbeke et al. [2011]; Schellenberg et al. [2011]).

The phylogeny of *P. putida* UW4 was described on the basis of 16S rRNA genes and a multilocus approach that used four concatenated housekeeping genes (16S rRNA, *gyrB, rpoD and rpoB*). *P. putida* UW4 was found to be closely related to *P. fluorescens* rather *P. putida* strains including *P. putida* LS46 (Duan et al. [2013]). It confirmed our observations on the phylogeny of *P. putida* UW4 based on the *cpn60* gene that *P. putida* UW4 was clustered with *P. fluorescens.* Earlier, Loper et al. ([2012]) on the basis of ten concatenated house keeping genes, showed similar phylogenetic relationships among *Pseudomonas* species. The *cpn60* gene phylogeny placed the ten sequenced *P. putida* strains and *P. entomophila* in the same clade. The phylogeny of the newly isolated *P. putida* LS46 based on *cpn60* differentiated this strain from other closely related *P.putida* strains, and based on both the 16S rRNA and *cpn60* analyses, *P. putida* LS46 was closely related to type strain *P. putida* KT2440. No plasmids have been detected in *P. putida* LS46, but two plasmids were present in *P. putida* ND6 and one plasmid was identified in *P. putida* DOT-T1E (Li et al. [2002]; Udaondo et al. [2012]).

Variation in genome content is thought to be a key factor in the evolution of bacteria and variation in genome arrangement may also improve the fitness of the bacteria (Silby et al. [2011a,b]). Presence of duplicated genes like rRNA operons, multiple transposons, insertion sequences, prophages and genomic islands leads to genome rearrangements in bacteria that contribute to evolution (Tillier and Collins [2000]; Klockgether et al. [2010]). Rearrangements in genome are not random, but predominantly occur at end-points either at the origin or at the terminus of replication (Eisen et al. [2000]).

Overall, strains of *P. putida* isolated from different geographical regions and from varied ecological niches had high similarity in genome structure and functions (Molina et al. [2011]; Li et al. [2012]; Nelson et al. [2002]; Wu et al. [2011]). All the *P. putida* genomes had higher level of genome similarity, but differed from other on the basis of insertion sequence, presence of prophage and genomic islands. However, Dot plot analyses of the *P. putida* strain genomes identified a number of rearrangements, which are possibly due to presence of these genetic elements, such as the presence of ISPa42 in *P. putida* LS46, which originated from *P. aeruginosa* DK3. ISPa42 may be associated with the acquisition of genes for heavy metal resistance.

In comparison to *P. putida* LS46, genome inversions were observed in *P. putida* BIRD-1 and *P. putida* W619 at the origin and terminus of replication in *P. putida* S16 and *P. putida* ND6, respectively. These observations strongly support the contention that bacterial genomes are not static and significant variations are observed even among strains within the same species. These variations are the result of genome deletions and/or gene acquisitions by horizontal gene transfer of elements such as transposon and genomic islands (Hacker and Carniel [2001], Mackiewicz et al. [2001]).

*P. putida* LS46 and *P. putida* DOT-T1E had a maximum sequence identity of 97.7%, but their genome arrangements were very diverse (Figure 2). It appears that the genome of *P. putida* DOT-T1E has undergone a number of rearrangements without losing any major function. *P. putida* DOT-T1E was isolated as toluene degrading species, and has acquired genes for toluene degradation that are absent in *P. putida* LS46. Using BLAST analysis 81.84-93.99% genes were identified which were shared among the 10 *P. putida* strains, while the rest of the genes were unique to the strain. Ballerstedt et al. ([2007]), using specific gene probe of *P. putida* KT2440 in a microarray identified 67.8-100% identical genes in six *P. putida* strains. Acquisition of specific groups of genes conferred specialized functions to specific strains, but their core genomes were identical.

Three *P. putida* strains, KT2440, BIRD1, and UW4, were isolated from soil and developed as plant growth promoting bacteria. *P. putida* strains F1, ND6, DOT-T1E, S16, GB1, and W619 were identified as bioremediation agents on the basis of their capacity to degrade pollutants. *P. putida* strain LS46 was isolated from wastewater for PHA production. This strain was able to use glucose, glycerol, fatty acids, and waste fryer oil and accumulates PHAs to 20-58% of the cell dry weight. Like other *P. putida* strains, central metabolic pathways for utilization of these substrates were also present in *P. putida* LS46. The application based classification of *P. putida* as biocontrol, bioremediation, or PHA producer is not relevant because biocontrol agent BIRD-1, or bioremediation strains F1, ND6, and DOT-T1E also encode the genes for PHA synthesis and homology among these genes is very high. The most of the genes present in the manganese oxidizing strain *P. putida* GB-1 or the endophyte of poplar stain *P. putida* W619 are also present in *P. putida* LS46.

Complete genome sequence of *P. putida* LS46 was compared with *P. putida* strains isolated from different geographical regions and different niches. Two strains, *P. putida* ND6 and *P. putida* DOT-T1E isolated from China, and *P. putida* F1 isolated from USA, had the highest sequence identities to *P. putida* LS46, which was isolated in Canada. *P. putida* strains GB-1 isolated from USA, UW4 isolated in Canada, and S16 isolated from China, had low sequence identities with *P. putida* LS46. *P. putida* W619, which was isolated as an endophyte of poplar had only 87.1% sequence identity with the *P. putida* LS46 genome, while *P. putida* UW4 showed only 82.0% identity with *P. putida* LS46. Our earlier result on phylogeny of *P.putida* UW4 has clearly demonstrated that this strain is related to *P. fluorescens* rather than *P. putida*. The low genome identity of *P. putida* strains with *P. putida* UW4 is not surprising. High genome similarities among *P. putida* strains isolated from different regions indicate their common ancestry (Biello [2008]).

**Figure 6:**
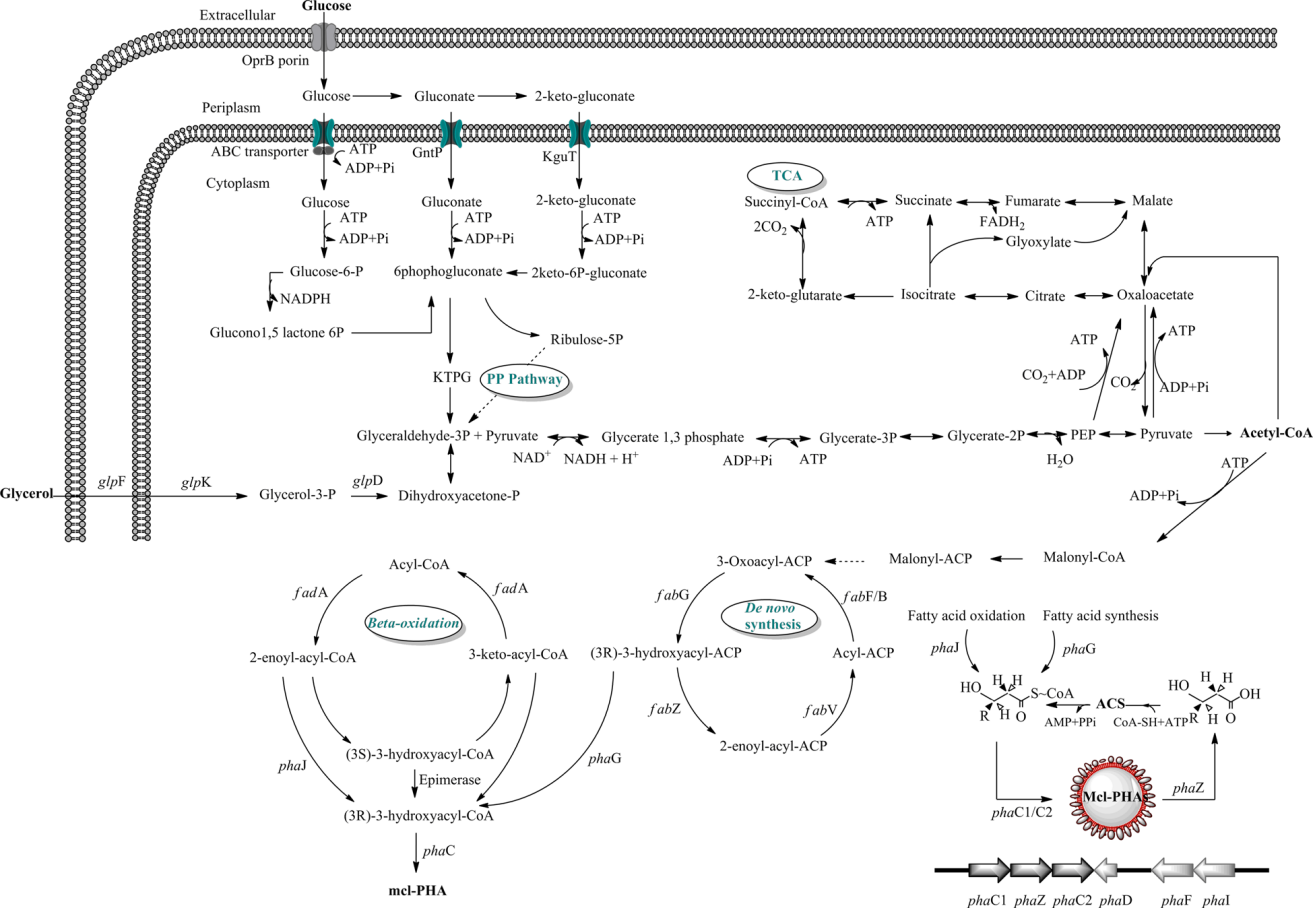
**Proposed PHA synthesis and degradation pathways for*P. putida*LS46.** Metabolic pathways involved carbon metabolism, fatty acid synthesis and degradation, and PHA synthesis are summarized. PHA can be synthesized from glucose, glycerol, or fatty acids via the PHA synthesis pathway.

*P. putida* LS46 shares a number of metabolic features with *P. putida* KT2440, *P. putida* F1, *P. putida* BIRD1, and *P. putida* GB-1, such as metabolism of aromatic compounds, manganese oxidation, root colonization, and PHAs production. All *P. putida* genomes have the ability to synthesize PHA irrespective of their applications, either as biocontrol agents or bioremediation agents. *P. putida* LS46 differs from other *P. putida* strains in number of dioxygenase genes, TonB dependent receptors, transferases, hydrolases, dehydrogenases and transferases. *P. putida* LS46, like other *P. putida* strains metabolize glucose, glycerol, and fatty acid by glycolysis, the tricarboxylic acid cycle, the pentose pathway, and β oxidation (Figure 6). At least three different metabolic pathways provide the precursors for the synthesis of PHAs. (i) Fatty acid *de novo* biosynthesis is the main route during growth on carbon sources that are metabolized to acetyl-CoA, like glucose, gluconate, glycerol etc. (ii) β-oxidation is the main pathway for PHAs production when fatty acids are used as carbon source, (iii) chain elongation reactions in which acetyl-CoA moieties are condensed to 3-hydroxyacyl-CoA is involved in the PHA synthesis when small chain length fatty acids like C6 and C7 are used. Intermediates of fatty acid *de novo* synthesis (3 hydroxylacyl-ACP) as well as fatty acid degradation pathways (3-hydroxylacyl-CoA) are used as precursors for PHAs production. Rehm et al. ([1998]) identified a link between fatty acid degradation and fatty acid synthesis by confirming the PHAs biosynthesis in β-oxidation defection mutants (*fadB*). This enzyme converts 3hydroxyacyl-ACP to 3-hydroxyacyl-CoA, which is a substrate for polymerization to PHAs. Glucose and glycerol are transported and metabolized to acetyl-CoA, which is further used for production of various fatty acids using fatty acid biosynthesis (*fab*) genes. Fatty acids or waste fryer oil containing long chain fatty acids (C16 and C18) are utilized by β-oxidation and intermediates are used for PHAs production (Wang et al. [2012]). Manipulation of fatty acid synthesis and degradation genes is known to improve PHAs production with altered monomer composition (Fiedler et al. [2002]). PHAs production in *P.putida* is a part of central metabolic pathway and it was evident from high level of identity among PHAs production proteins and proteins of feeding pathways like fatty acid biosynthesis and degradation.

*P. putida* LS46 does not match, however, the metabolic diversity of either *P. putida* F1 or *P. putida* DOT-T1E, which encode genes for degradation of toluene, cymene, and 3-hydroxyphenylpropionate, or *P. putida* ND6, which encodes genes for naphthalene degradation. These genes may have been acquired during the evolution process. A number of transposons and insertion sequences were present in *P. putida* genomes indicating their labile nature. ISPpu10, a transposon present in REP sequence of *P. putida* KT2440 has been implicated in genome rearrangement (Ramos-Gonzalez et al. [2006]). But this transposon was absent from all *P. putida* strain except *P. putida* S16. Mg (II) oxidation (*P. putida* GB1), pyoverdine production, aromatic/aliphatic sulphonate degradation (*P. putida* KT2440), and polyhydroxyalkanoate production (*P. putida* strains KT2440 and LS46) are core genes that are present in all *P. putida* strains whether these strains were isolated as biocontrol agents or as bioremediation agents (Wu et al. [2011]). The genome of *P. putida* LS46 had a majority of the genes involved in plant growth promotion even it was not isolated as plant growth promoter and in this regards it was similar to *P. putida* KT2440 and *P. putida* BIRD1. Several unique genes were identified in the *P. putida* LS46 genome. The presence of prophages, insertion sequences, and genomic islands in different genomes indicate that the *P. putida* genome is not static, but changes by acquiring new genes from related and unrelated species.

## Competing interests

The authors declare that they have no competing interests, financial or otherwise.

## Authors' contributions

The nucleotide sequence alignments, genome analyses, and preparation of the manuscript draft were carried out by PKS. JF assisted PKS with the genome analyses. The genome assembly and annotation, and uploading of files to IMG were carried out by XLZ, who was supervised by BF. RS and DBL are the co-PIs for the Genome Canada funded research program, and DBL served as PKS’s direct supervisor. DBL also prepared the final draft of the manuscript for submission. All authors read and approved the final manuscript.

## Additional file

## Supplementary Material

Additional file 1: Table S1. Occurrence of IS elements in different *P.putida* strains. **Table S2**: Predicted prophages in *P. putida* genomes. **Table S3**: Homology of house keeping genes encoded in the genomes of different *P. putida* strains. **Table S4**: Homology of house keeping genes encoded in the genomes of different *P. putida* strains. **Table S5**: Occurrence of different dioxygenases encoded in the *P.putida* LS46 genome and their homologues encoded in the genomes of other *P. putida* strains. **Table S6**: Presence of genes encoded in the *P. putida* KT2440 genome associated with different metabolic pathways in other *P.putida* strains. **Table S7**: Presence of genes encoded by the *P. putida* F1 genome involved in aromatic compound degradation and their homologues encoded in the genomes of other *P. putida* strains. **Table S8**: Occurrence of heavy metal tolerance genes encoded by *P. putida* W619 in different *P. putida* strains. **Table S9**: Identification of TonB receptors in *P. putida* LS46 and their homologues in other *P. putida* strains. Click here for file
