# Supplementary material for: Genome features of Pseudomonas putida LS46, a novel polyhydroxyalkanoate producer and its comparison with other P. putida strains
Source: AMB Express. 2014 May 22;4:37. doi: 10.1186/s13568-014-0037-8 (PMC4230813; doi:10.1186/s13568-014-0037-8)
Supplement: Additional file 1: Table S1. — Occurrence of IS elements in different P.putida strains. Table S2: Predicted prophages in P. putida genomes. Table S3: Homology of house keeping genes encoded in the genomes of different P. putida strains. Table S4: Homology of house keeping genes encoded in the genomes of different P. putida strains. Table S5: Occurrence of different dioxygenases encoded in the P.putida LS46 genome and their homologues encoded in the genomes of other P. putida strains. Table S6: Presence of genes encoded in the P. putida KT2440 genome associated with different metabolic pathways in other P.putida strains. Table S7: Presence of genes encoded by the P. putida F1 genome involved in aromatic compound degradation and their homologues encoded in the genomes of other P. putida strains. Table S8: Occurrence of heavy metal tolerance genes encoded by P. putida W619 in different P. putida strains. Table S9: Identification of TonB receptors in P. putida LS46 and their homologues in other P. putida strains. [file s13568-014-0037-8-S1.docx]

**Additional file 1, AMB Express**

**Genome features of *Pseudomonas putida* LS46, a novel polyhydroxyalkanoate producer, and comparison with other *P. putida* strains.**

Parveen K. Sharma^1^, Jilagamazhi Fu^1^, Xangli Zhang^2^, Brian Fristensky^2^, Richard Sparling^3^ and David B. Levin^1^*

^1^Department of Biosystems Engineering, ^2^Department of Plant Science, and ^3^Department of Microbiology University of Manitoba, Winnipeg, MB, Canada, R3T 2N2

* Corresponding Author: David B. Levin; E-mail: [david.levin@umanitoba.ca](mailto:david.levin@umanitoba.ca); Telephone: (204) 474-7429; Fax: (204) 474-7512

**Table S1.** Occurrence of IS elements in different *P.putida* strains.

| **Strain** | **IS element** | **Parent in IS database** | **Size in Parent** | **Copy Number** | **Size of identified IS** | **Homologous Region**  **(% Homology)** | **Coordinates** |
| --- | --- | --- | --- | --- | --- | --- | --- |
| LS46 | ISPa42 | Tn3 | 16941 | 1 | 12176 | 1-12176 (98%) | 1030749-1042924 |
|  |  |  | 16941 | 1 | 4765 | 12177-16941 (97%) | 1006064-1010827 |
|  | ISPu9 | IS110 | 2043 | 1 | 1697 | 1-1697 (98%) | 4999844-5001540 |
|  |  |  | 2043 | 1 | 1697 | 1-1697(98%) | 4823543-4825223 |
|  |  |  | 2043 | 1 | 1697 | 1-1697(98%) | 4345794-4347490 |
|  | ISPa41 | IS5 | 1168 | 1 | 1168 | 1-1168(99%) | 3518995-3520162 |
|  | ISPs1 | IS5 | 910 | 1 | 907 | 1-907 (84%) | 3517130-3517189 |
|  |  |  | 910 | 1 | 907 | 1-907 (84%) | 3528841-3529747 |
| DOT-T1E | ISPpu13 | IS66 | 2370 | 1 | 1989 | 1-1989 (97%) | 3320265-3322253 |
|  | ISPpu14 | IS66 | 2383 | 1 | 2350 | 1-2350 (92%) | 5295968-5298317 |
|  | ISPen2 | IS3 | 1232 | 1 | 1186 | 47-1232 (92%) | 3372698-3373883 |
|  | ISPpu22 | IS3 | 1232 | 1 | 1186 | 47-1232 (92%) | 3372698-3373883 |
| ND6 | ISPpu14 | IS66 | 2383 | 2 | 2383  2383 | 1-2383 (93%)  1-2383 (93%) | 2564065-2566447  4900919-4903301 |
|  | ISPpu16 | IS1182 | 1677 | 2 | 1520  1520 | 1-1520 (94%)  1-1520 (94%) | 397621-399143  5004671=5006135 |
|  | ISPa41 | IS5 | 1168 | 1 | 1168 | 1-1168 (99%) | 1883861-1884969 |
|  | IS2000 | IS30 | 1186 | 2 | 603  583  790 | 1-603 (99%)  604-1186 (99%)  277-1065 (88%) | 339190-339730  336697-337279  377410-378199 |
|  | IS1382 | IS5 | 1093 | 1 | 1093 | 1-1093(97%) | 376318-377349 |
|  | ISPs1 | ISL3 | 910 |  | 907 | 1-907 (84%) | 3517130-3518036 |
| BIRD1 | ISPpu13 | IS66 | 2370 | 1 | 1986 | 1-1986 (96%) | 785480-787460 |
|  | ISPpu9 | IS110 | 2043 | 1 | 1694 | 1-1694 (97%) | 1666036-1667720 |
|  | ISPpu15 | IS66 | 2041 | 1 | 465  549  166 | 36-500 (84%)  853-1401 (84%)  1806-1971 (84%) | 786433-786969  786068-786616  785498-785663 |
| F1 | IS2000 | IS5 | 1186 | 1 | 1186 | 1-1186 (98%) | 1525767-1526952 |
|  | ISPen2 | IS3 | 1232 | 1 | 809 | 424-1232 (90%) | 479560-480368 |
|  | ISPpu22 | IS3 | 1232 | 1 | 809 | 424-1232 (90%) | 479560-480368 |
|  | ISPsy24 | IS5 | 1235 | 1 | 548 | 539-1086 (91%) | 480907-481453 |
| W619 | ISPPu12 | ISL3 | 3372 | 1 | 3372 | 1-3372 (100%) | 2602742-2606113 |
|  | ISPpu14 | IS66 | 2383 | 1 | 2383 | 1-2383 (92%) | 40282-42664 |
|  | IS1386 | ISL3 | 1771 | 1 | 1325 | 1-1325 (100%) | 2604789-2606613 |
|  | ISPa27 | IS256 | 1361 | 1 | 1360 | 1-1360 (99%) | 3653998-3655359 |
|  | ISPa40 | Tn3 | 6592 | 1 | 2209  1116 | 4190-6398 (88%)  3020-4135 (87%) | 2598041-2600249  2600304-2601419 |
|  | ISPst2 | ISL3 | 2984 | 1 | 1241 | 1745-2984 (99%) | 2604874-2606113 |
|  | ISPpu16 | IS1182 | 1677 | 1 | 1520 | 1-1520 (94%) | 3656408-3657930 |
|  | ISPpu18 | IS5 | 1192 | 1 | 1192 | 1-1192 (97%) | 3697397-3698528 |
|  | IS1382 | IS30 | 1093 | 1 | 1093 | 1-1093 (97%) | 2219231-2220321 |
|  | ISPst9 | ISL3 | 2472 | 1 | 1348 | 1125-2444 (90%) | 2604766-2606113 |
|  | ISThi1 | IS5 | 1191 | 1 | 1061 | 65-1125 (87%) | 3697402-3698462 |
|  | ISAch1 | IS5 | 1192 | 1 | 1023 | 67-1089 (87%) | 3697402-3698462 |
|  | ISPre2 | IS5 | 1190 | 1 | 994 | 65-1056 (87%) | 3697449-3698524 |
|  | ISPst7 | IS5 | 1192 | 1 | 1135 | 54-1188 (85%) | 3697449-3698524 |
|  | ISPst5 | IS5 | 1191 | 1 | 1078 | 5-1082 (86%) | 3697341-3698418 |
|  | IS1384 | IS5 | 1178 | 1 | 994 | 59-1052 (87%) | 3697702-3698395 |
|  | ISPre1 | IS5 | 1192 | 1 | 1120 | 7-1126 (85%) | 3697343-3698422 |
|  | ISPps11 | IS5 | 1188 | 1 | 994 | 65-1056 (85%) | 3697402-3698361 |
|  | ISPa38 | Tn5 | 6455 | 1 | 968 | 5292-6250 (86%) | 2598051-2599010 |
|  | ISPpu21 | IS5 | 1190 | 1 | 950 | 65-1014 (86%) | 3697402-3698351 |
|  | ISPsy2 | IS5 | 1194 | 1 | 993 | 68-1060 (83%) | 3697402-3698361 |
|  | ISPa26 | IS26 | 1193 | 1 | 953 | 174-1126 (82%) | 3697402-3698354 |
| KT2440 | ISPpu14 | IS66 | 2383 | 6 | 2383  2383  2383  2383  2383  2383 | 1-2383 (100%)  1-2383 (93%)  1-2383 (93%)  1-2383 (93%)  1-2383 (93%)  1-2383 (93%) | 6152898-6155280  5036091-5038473  3969263-3971645  4486295-4488677  5033143-5035525  4473963-4476350 |
|  | ISPPu13 | IS66 | 2370 | 2 | 2370  2370 | 1-2370 (100%)  1-2370 (100%) | 4493333-4495702  3522822-3525191 |
|  | ISPpu15 | IS66 | 2043 | 9 | 2040 | 1-2040 (100%)  1-2040 (99%)  1-2040 (100%)  1-2040 (100%)  1-2040 (100%)  1-2040 (99%)  1-2040 (99%)  1-2040 (99%)  1-2040 (99%) | 5401745-5407384  4625922-4627961  4536200-4538239  744919-746958  5222580-5224610  12964831298522  1439998-1442037  4073696-4075735  3826259-3828298 |
| UW4 | ISPpu2 |  | 1131 | 1 | 772 | 1-772 (92%) | 41223228-41223996 |
|  | ISPa11 |  | 928 | 1 | 636 | 353-928 (85%) | 3912756-3913389 |
|  | ISCfri |  | 1617 | 7 | 785 | 208-928 (81%) | 2483627-2484411 |
|  | ISPsp5 |  | 1617 | 7 | 1169 | 82-1247 (79%) | 2588254-2589422 |
|  | ISPpu |  | 910 |  | 907 | 1-907 (84%) | 3528841=3529747 |
|  | IS2000 |  | 1186 |  | 348 | 84-431 (85%) | 3519756-3520103 |
|  |  |  | 1186 |  | 275 | 480-754 (79%) | 3519443-3519707 |
|  |  |  | 1186 |  | 456 | 37-492 (82%) | 3528877-3529332 |
|  | IsPen2 |  | 1232 |  | 225 | 480-754 (90%) | 4104639-4104863 |
|  |  |  | 1232 |  | 155 | 375-529 (87%) | 4104474-4104628 |
|  | IsPst8 |  | 910 |  | 456 | 37-492 (82%) | 3517166-3517621 |
|  |  |  | 910 |  | 456 | 37-492 (82%) | 3528877-3529332 |
|  | ISPu22 |  | 1232 |  | 158 | 376-533 (95%) | 4104534-4104632 |
|  |  |  | 1232 |  | 119 | 376-533 (95% | 4106433-4104863 |
|  | ISPu14 |  | 2383 |  | 355 | 1986-2340 (83%) | 3515542-3515841 |
|  |  |  | 2383 |  | 234 | 417-650 (87%) | 3429853-3530086 |
|  |  |  | 2383 |  | 387 | 1165-1551 (82%) | 3516331-3516717 |
|  |  |  | 2383 |  | 199 | 2144-2341 (86%) | 983977-984174 |
|  |  |  | 2383 |  | 95 | 1-95 (95%) | 948664-948758 |
|  |  |  | 2383 |  | 200 | 1672-1871 (84%) | 3516011-3516210 |
|  |  |  | 2383 |  | 135 | 33-167 (82%) | 3530339-3530459 |
|  |  |  | 2383 |  | 152 | 889-1040 (80%) | 35166842-3516993 |
| GB-1 | ISPpu22 | IS3 | 1232 | 4 | 1232 | 1-1232 (100%) | 1823889-1825120 |
|  |  |  |  |  | 1232 | 1-1232 (100%) | 3324260-3325491 |
|  |  |  |  |  | 1232 | 1-1232 (100%) | 4234589-4235820 |
|  |  |  |  |  | 1232 | 1-1232 (100%) | 6056867-6058098 |
|  | ISPa41 | IS5 | 1168 | 1 | 1168 | 1-1168 (99%) | 5390819-5391986 |
|  | ISPen2 | IS3 | 1232 | 4 | 1232 | 1-1232 (90% | 1823889-1825120 |
|  |  |  | 1232 |  | 1232 | 1-1232 (90%) | 3324260-3325491 |
|  |  |  | 1232 |  | 1232 | 1-1232 (90%) | 4234589-4235820 |
|  |  |  | 1232 | 4 | 1232 | 1-1232 (90%) | 6056867-6058098 |
|  | ISPpu15 | IS66 | 2041 | 4 | 2026 | 17-2041(82%) | 595456-597477 |
|  |  |  |  |  | 2026 | 17-2041(82%) | 5360670-5362691 |
|  |  |  |  |  | 2026 | 17-2041(82%) | 1941516-1943537 |

**Table S2.** Predicted prophages in *P. putida* genomes.

| **Strain** | **Region** | **Region length** | **Completeness*** | **Score** | **CDS** | **Region position** | **Possible phage** | **%GC** |
| --- | --- | --- | --- | --- | --- | --- | --- | --- |
| BIRD1 | [1](http://phast.wishartlab.com/cgi-bin/change_detail_html.cgi?num=1382718855#1) | 42.7Kb | Questionable | 90 | 40 | [3114451-3157233](http://phast.wishartlab.com/cgi-bin/get_region_DNA.cgi?num=1382718855&number=1) | PHAGE_Pseudo_vB_PaeS_PMG1 | 59.66% |
| LS46 | [1](http://phast.wishartlab.com/cgi-bin/change_detail_html.cgi?num=1383072662#1) | 49.6Kb | Intact | 150 | 61 | [2509194-2558873](http://phast.wishartlab.com/cgi-bin/get_region_DNA.cgi?num=1383072662&number=1) | PHAGE_Pseudo_vB_PaeS_PMG1 | 59.16% |
|  | [2](http://phast.wishartlab.com/cgi-bin/change_detail_html.cgi?num=1383072662#2) | 6.7Kb | Incomplete | 60 | 7 | [3924740-3931529](http://phast.wishartlab.com/cgi-bin/get_region_DNA.cgi?num=1383072662&number=2) | PHAGE_Stx2_converting_1717 | 51.74% |
| DOT-T1E | [1](http://phast.wishartlab.com/cgi-bin/change_detail_html.cgi?num=1382718335#1) | 32.2Kb | Incomplete | 40 | 16 | [1482573-1514844](http://phast.wishartlab.com/cgi-bin/get_region_DNA.cgi?num=1382718335&number=1) | PHAGE_Pseudo_PAJU2 | 60.62% |
|  | [2](http://phast.wishartlab.com/cgi-bin/change_detail_html.cgi?num=1382718335#2) | 35.1Kb | Intact | 100 | 49 | [1515113-1550262](http://phast.wishartlab.com/cgi-bin/get_region_DNA.cgi?num=1382718335&number=2) | PHAGE_Klebsi_JD001 | 59.42% |
|  | [3](http://phast.wishartlab.com/cgi-bin/change_detail_html.cgi?num=1382718335#3) | 39.2Kb | Intact | 150 | 48 | [1567610-1606836](http://phast.wishartlab.com/cgi-bin/get_region_DNA.cgi?num=1382718335&number=3) | PHAGE_Escher_HK639 | 61.34% |
| F1 | [1](http://phast.wishartlab.com/cgi-bin/change_detail_html.cgi?num=1382477831#1) | 50.7Kb | Intact | 120 | 49 | [3778059-3828816](http://phast.wishartlab.com/cgi-bin/get_region_DNA.cgi?num=1382477831&number=1) | PHAGE_Pseudo_PAJU2 | 59.92% |
|  | [2](http://phast.wishartlab.com/cgi-bin/change_detail_html.cgi?num=1382477831#2) | 58.4Kb | Intact | 100 | 58 | [4600889-4659292](http://phast.wishartlab.com/cgi-bin/get_region_DNA.cgi?num=1382477831&number=2) | PHAGE_Pseudo_D3 | 60.49% |
| GB1 | 1 | 42.2Kb | Intact | 150 | 31 | [1336655-1378929](http://phast.wishartlab.com/cgi-bin/get_region_DNA.cgi?num=1382474930&number=1) | PHAGE_Vibrio_vB_VpaM_MAR | 60.81% |
|  | 2 | 66.9Kb | Intact | 120 | 55 | [1929732-1996699](http://phast.wishartlab.com/cgi-bin/get_region_DNA.cgi?num=1382474930&number=2) | PHAGE_Escher_TL_2011b | 60.24% |
|  | 3 | 41.6Kb | Intact | 140 | 56 | [3820676-3862321](http://phast.wishartlab.com/cgi-bin/get_region_DNA.cgi?num=1382474930&number=3) | PHAGE_Cronob_phiES15 | 60.26% |
| KT2440 | [1](http://phast.wishartlab.com/cgi-bin/change_detail_html.cgi?num=1382473875#1) | 32.8Kb | Intact | 130 | 32 | [1748226-1781069](http://phast.wishartlab.com/cgi-bin/get_region_DNA.cgi?num=1382473875&number=1) | PHAGE_Pseudo_vB_PaeS_PMG1 | 61.12% |
|  | [2](http://phast.wishartlab.com/cgi-bin/change_detail_html.cgi?num=1382473875#2) | 27.6Kb | Incomplete | 20 | 9 | [2831042-2858715](http://phast.wishartlab.com/cgi-bin/get_region_DNA.cgi?num=1382473875&number=2) | PHAGE_Bacill_phiNIT1 | 57.56% |
|  | [3](http://phast.wishartlab.com/cgi-bin/change_detail_html.cgi?num=1382473875#3) | 36Kb | Intact | 150 | 41 | [3411566-3447568](http://phast.wishartlab.com/cgi-bin/get_region_DNA.cgi?num=1382473875&number=3) | PHAGE_Vibrio_vB_VpaM_MAR | 61.16% |
|  | [4](http://phast.wishartlab.com/cgi-bin/change_detail_html.cgi?num=1382473875#4) | 40.3Kb | Intact | 150 | 47 | [4375283-4415646](http://phast.wishartlab.com/cgi-bin/get_region_DNA.cgi?num=1382473875&number=4) | PHAGE_Entero_SfV | 60.13% |
| ND6 | [1](http://phast.wishartlab.com/cgi-bin/change_detail_html.cgi?num=1382719498#1) | 17.3Kb | Intact | 100 | 27 | [1888422-1905754](http://phast.wishartlab.com/cgi-bin/get_region_DNA.cgi?num=1382719498&number=1) | PHAGE_Entero_P4 | 55.63% |
|  | [2](http://phast.wishartlab.com/cgi-bin/change_detail_html.cgi?num=1382719498#2) | 7.9Kb | Incomplete | 50 | 9 | [2992080-2999994](http://phast.wishartlab.com/cgi-bin/get_region_DNA.cgi?num=1382719498&number=2) | PHAGE_Burkho_phi52237 | 54.47% |
|  | [3](http://phast.wishartlab.com/cgi-bin/change_detail_html.cgi?num=1382719498#3) | 40.8Kb | Intact | 150 | 47 | [4096036-4136842](http://phast.wishartlab.com/cgi-bin/get_region_DNA.cgi?num=1382719498&number=3) | PHAGE_Vibrio_vB_VpaM_MAR | 59.81% |
|  | 4 | 25.7Kb | Incomplete | 40 | 33 | [4888680-4914427](http://phast.wishartlab.com/cgi-bin/get_region_DNA.cgi?num=1382719498&number=4) | PHAGE_Pseudo_F10, | 58.02% |
| S16 | [1](http://phast.wishartlab.com/cgi-bin/change_detail_html.cgi?num=1382992406#1) | 60.4Kb | Intact | 150 | 53 | [1353421-1413835](http://phast.wishartlab.com/cgi-bin/get_region_DNA.cgi?num=1382992406&number=1) | PHAGE_Entero_SfV | 60.32% |
|  | [2](http://phast.wishartlab.com/cgi-bin/change_detail_html.cgi?num=1382992406#2) | 24.7Kb | Intact | 150 | 29 | [1959736-1984471](http://phast.wishartlab.com/cgi-bin/get_region_DNA.cgi?num=1382992406&number=2) | PHAGE_Vibrio_vB_VpaM_MAR, | 63.16% |
|  | [3](http://phast.wishartlab.com/cgi-bin/change_detail_html.cgi?num=1382992406#3) | 24.9Kb | Incomplete | 50 | 34 | [2762204-2787150](http://phast.wishartlab.com/cgi-bin/get_region_DNA.cgi?num=1382992406&number=3) | PHAGE_Acinet_Bphi_B1251 | 61.05% |
|  | [4](http://phast.wishartlab.com/cgi-bin/change_detail_html.cgi?num=1382992406#4) | 21.1Kb | Incomplete | 20 | 19 | [3708833-3730016](http://phast.wishartlab.com/cgi-bin/get_region_DNA.cgi?num=1382992406&number=4) | PHAGE_Ectoca_siliculosus_virus1 | 60.68% |
| W619 | [1](http://phast.wishartlab.com/cgi-bin/change_detail_html.cgi?num=1382468846#1) | 42.8Kb | Intact | 140 | 59 | [1437048-1479934](http://phast.wishartlab.com/cgi-bin/get_region_DNA.cgi?num=1382468846&number=1) | PHAGE_Pseudo_vB_PaeS_PMG1 | 60.27% |
|  | [2](http://phast.wishartlab.com/cgi-bin/change_detail_html.cgi?num=1382468846#2) | 10.8Kb | Incomplete | 30 | 9 | [3034880-3045683](http://phast.wishartlab.com/cgi-bin/get_region_DNA.cgi?num=1382468846&number=2) | PHAGE_Cronob_ENT39118 | 60.46% |
|  | [3](http://phast.wishartlab.com/cgi-bin/change_detail_html.cgi?num=1382468846#3) | 43Kb | Intact | 150 | 55 | [4352627-4395680](http://phast.wishartlab.com/cgi-bin/get_region_DNA.cgi?num=1382468846&number=3) | PHAGE_Pseudo_F10 | 59.42% |
|  | [4](http://phast.wishartlab.com/cgi-bin/change_detail_html.cgi?num=1382468846#4) | 23.3Kb | Questionable | 90 | 26 | [4452059-4475372](http://phast.wishartlab.com/cgi-bin/get_region_DNA.cgi?num=1382468846&number=4) | PHAGE_Pseudo_phiCTX | 60.45% |

**Table S3.** Homology of house keeping genes encoded in the genomes of different *P. putida* strains.

| **Locus tag** | **Gene** | **Product name** | **% Homology** | | | | | | | | |
| --- | --- | --- | --- | --- | --- | --- | --- | --- | --- | --- | --- |
|  |  |  | **UW4** | **F1** | **ND6** | **BIRD1** | **DOT-T1E** | **GB1** | **KT2440** | **W619** | **S16** |
| 019951 | *dnaX* | DNA polymerase III, | 92.73 | 99.13 | 96.3 | 95.52 | 96.55 | 92.49 | 96.1 | 84.55 | 87.93 |
| 000330 | uvrB | Excision nuclease subunit ß | 90.90 | 100 | 99.68 | 99.85 | 100 | 99.11 | 99.85 | 92.62 | 98.36 |
| 016679 | atpD | ATP synthase ß subunit | 95.41 | 100 | 100 | 100 | 100 | 99.56 | 100 | 96.94 | 99.56 |
| 021536 | aspS | Aspartate tRNA synthetase | 91.20 | 99.83 | 99.83 | 99.83 | 99.15 | 99.66 | 99.83 | 99.15 | 98.98 |
| 002847 | cysS | Cysteine tRNA synthetase | 87.61 | 98.91 | 99.13 | 99.57 | 99.13 | 97.83 | 98.91 | 97.17 | 98.48 |
| 012340 | uvrC | Exonuclease ABC, subunit C | 85.17 | 100 | 100 | 99.67 | 99.83 | 98.80 | 99.51 | 96.21 | 98.19 |
| 021516 | ruvB | Holliday junction helicase subunit A | 86.21 | 99.43 | 99.71 | 99.71 | 99.71 | 98.28 | 99.71 | 96.84 | 97.41 |
| 022086 | metG | Methionine tRNA synthetase | 89.02 | 99.71 | 99.85 | 99.71 | 99.85 | 98.67 | 99.41 | 96.42 | 98.23 |
| 006251 | dnaB | Replicative DNA helicase | 90.11 | 100 | 100 | 99.78 | 100 | 98.49 | 99.78 | 98.28 | 98.92 |
| 007106 | dnaJ | Chaperone with dnaK | 86.84 | 100 | 100 | 98.78 | 100 | 99.73 | 98.67 | 98.66 | 98.93 |
| 017909 | rho | Transcription termination factor | 97.61 | 100 | 100 | 100 | 100 | 100 | 100 | 100 | 100 |
| 021591 | proS | Proline tRNA synthetase | 87.93 | 99.82 | 99.82 | 99.30 | 99.82 | 97.55 | 99.47 | 97.57 | 97.72 |
| 014719 | recA | DNA strand exchange/ renaturation | 88.35 | 100 | 100 | 100 | 100 | 98.31 | 100 | 99.09 | 98.87 |
| 023893 | rpoA | RNA polymerase alpha subunit | 99.10 | 100 | 100 | 100 | 100 | 100 | 100 | 100 | 100 |
| 014639 | eno | Enolase | 92.79 | 100 | 100 | 100 | 100 | 99.53 | 100 | 99.53 | 99.3 |
| 022046 | lig | DNA ligase | ND | 96.04 | 95.80 | 95.80 | 95.80 | 87.74 | 95.32 | 84.48 | 87.74 |
| 007236 | pgi | Glucose phosphate isomerase | 89.35 | 99.16 | 99.16 | 99.16 | 99.16 | 94.51 | 97.89 | 86.08 | 91.56 |
| 023643 | dxs | Deoxyxylose phosphate synthase | 89.08 | 100 | 100 | 99.84 | 99.84 | 99.21 | 99.68 | 99.57 | 98.42 |
| 007091 | recN | Recombination/repair protein | 84.17 | 100 | 99.82 | 99.46 | 99.82 | 99.67 | 99.46 | 94.97 | 97.13 |
| 013648 | groEL | Chaperonin Hsp60 | 88.21 | 100 | 100 | 100 | 100 | 98.50 | 99.82 | 98.35 | 99.27 |
| 024688 | glyA | Serine hydroxymethyltransferase | 96.64 | 100 | 100 | 100 | 100 | 85.34 | 100 | 98.56 | 99.52 |
| 014003 | lepA | GTP binding elongation factor | 91.82 | 99.83 | 99.83 | 99.83 | 99.67 | 99.16 | 99.83 | 98.83 | 98.33 |
| 013403 | trpS | Tryptophan tRNA synthetase | 82.25 | 98.20 | 98.20 | 97.31 | 97.98 | 96.19 | 97.76 | 93.50 | 96.41 |
| 005196 | argS | Arginine tRNA synthetase | 87.26 | 100 | 99.83 | 97.31 | 97.98 | 96.81 | 99.48 | 96.99 | 98.1 |
| 014174 | ffh | GTP binding export factor | 93.35 | 100 | 100 | 100 | 100 | 99.78 | 100 | 99.34 | 99.13 |
| 011905 | serS | Serine tRNA synthetase | 88.24 | 99.77 | 99.77 | 99.53 | 99.77 | 98.36 | 99.77 | 97.65 | 98.36 |
| 013553 | ftsZ | Tubulin like division protein | 93.97 | 100 | 100 | 100 | 100 | 99.75 | 100 | 100 | 99.75 |
| 005806 | metK | Methionine adenosyltransferase | 90.91 | 100 | 100 | 100 | 100 | 100 | 100 | 98.74 | 98.74 |
| 016669 | atpA | ATP synthase F1, ß subunit | 95.14 | 99.61 | 99.61 | 100 | 99.61 | 99.81 | 99.61 | 98.64 | 98.44 |
| 005826 | pgk | Phosphoglycerate kinase | 93.02 | 100 | 100 | 98.97 | 100 | 99.74 | 99.74 | 97.67 | 97.83 |
| 009539 | tig | Trigger factor | 83.91 | 100 | 99.27 | 99.54 | 100 | 97.9 | 99.77 | 95.19 | 95.98 |
| 016619 | thdF/trmE | GTP binding, tRNA modification | 85.53 | 99.78 | 99.56 | 98.78 | 99.78 | 98.25 | 99.78 | 96.93 | 97.56 |

**Table S4.** Homology of house keeping genes encoded in the genomes of different *P. putida* strains.

| **Locus tag** | **Gene** | **Product name** | **% Homology** | | | | | | | | |
| --- | --- | --- | --- | --- | --- | --- | --- | --- | --- | --- | --- |
|  |  |  | **UW4** | **F1** | **ND6** | **BIRD-1** | **DOT-T1E** | **GB1** | **KT2440** | **W619** | **S16** |
| 019951 | *dnaX* | DNA polymerase III, | 92.73 | 99.13 | 96.3 | 95.52 | 96.55 | 92.49 | 96.1 | 84.55 | 87.93 |
| 000330 | uvrB | Excision nuclease subunit ß | 90.90 | 100 | 99.68 | 99.85 | 100 | 99.11 | 99.85 | 92.62 | 98.36 |
| 016679 | atpD | ATP synthase ß subunit | 95.41 | 100 | 100 | 100 | 100 | 99.56 | 100 | 96.94 | 99.56 |
| 021536 | aspS | Aspartate tRNA synthetase | 91.20 | 99.83 | 99.83 | 99.83 | 99.15 | 99.66 | 99.83 | 99.15 | 98.98 |
| 002847 | cysS | Cysteine tRNA synthetase | 87.61 | 98.91 | 99.13 | 99.57 | 99.13 | 97.83 | 98.91 | 97.17 | 98.48 |
| 012340 | uvrC | Exonuclease ABC, subunit C | 85.17 | 100 | 100 | 99.67 | 99.83 | 98.80 | 99.51 | 96.21 | 98.19 |
| 021516 | ruvB | Holliday junction helicase subunit A | 86.21 | 99.43 | 99.71 | 99.71 | 99.71 | 98.28 | 99.71 | 96.84 | 97.41 |
| 022086 | metG | Methionine tRNA synthetase | 89.02 | 99.71 | 99.85 | 99.71 | 99.85 | 98.67 | 99.41 | 96.42 | 98.23 |
| 006251 | dnaB | Replicative DNA helicase | 90.11 | 100 | 100 | 99.78 | 100 | 98.49 | 99.78 | 98.28 | 98.92 |
| 007106 | dnaJ | Chaperone with dnaK | 86.84 | 100 | 100 | 98.78 | 100 | 99.73 | 98.67 | 98.66 | 98.93 |
| 017909 | rho | Transcription termination factor | 97.61 | 100 | 100 | 100 | 100 | 100 | 100 | 100 | 100 |
| 021591 | proS | Proline tRNA synthetase | 87.93 | 99.82 | 99.82 | 99.30 | 99.82 | 97.55 | 99.47 | 97.57 | 97.72 |
| 014719 | recA | DNA strand exchange/ renaturation | 88.35 | 100 | 100 | 100 | 100 | 98.31 | 100 | 99.09 | 98.87 |
| 023893 | rpoA | RNA polymerase alpha subunit | 99.10 | 100 | 100 | 100 | 100 | 100 | 100 | 100 | 100 |
| 014639 | eno | Enolase | 92.79 | 100 | 100 | 100 | 100 | 99.53 | 100 | 99.53 | 99.3 |
| 022046 | lig | DNA ligase | ND | 96.04 | 95.80 | 95.80 | 95.80 | 87.74 | 95.32 | 84.48 | 87.74 |
| 007236 | pgi | Glucose phosphate isomerase | 89.35 | 99.16 | 99.16 | 99.16 | 99.16 | 94.51 | 97.89 | 86.08 | 91.56 |
| 023643 | dxs | Deoxyxylose phosphate synthase | 89.08 | 100 | 100 | 99.84 | 99.84 | 99.21 | 99.68 | 99.57 | 98.42 |
| 007091 | recN | Recombination/repair protein | 84.17 | 100 | 99.82 | 99.46 | 99.82 | 99.67 | 99.46 | 94.97 | 97.13 |
| 013648 | groEL | Chaperonin Hsp60 | 88.21 | 100 | 100 | 100 | 100 | 98.50 | 99.82 | 98.35 | 99.27 |
| 024688 | glyA | Serine hydroxymethyltransferase | 96.64 | 100 | 100 | 100 | 100 | 85.34 | 100 | 98.56 | 99.52 |
| 014003 | lepA | GTP binding elongation factor | 91.82 | 99.83 | 99.83 | 99.83 | 99.67 | 99.16 | 99.83 | 98.83 | 98.33 |
| 013403 | trpS | Tryptophan tRNA synthetase | 82.25 | 98.20 | 98.20 | 97.31 | 97.98 | 96.19 | 97.76 | 93.50 | 96.41 |
| 005196 | argS | Arginine tRNA synthetase | 87.26 | 100 | 99.83 | 97.31 | 97.98 | 96.81 | 99.48 | 96.99 | 98.1 |
| 014174 | ffh | GTP binding export factor | 93.35 | 100 | 100 | 100 | 100 | 99.78 | 100 | 99.34 | 99.13 |
| 011905 | serS | Serine tRNA synthetase | 88.24 | 99.77 | 99.77 | 99.53 | 99.77 | 98.36 | 99.77 | 97.65 | 98.36 |
| 013553 | ftsZ | Tubulin like division protein | 93.97 | 100 | 100 | 100 | 100 | 99.75 | 100 | 100 | 99.75 |
| 005806 | metK | Methionine adenosyltransferase | 90.91 | 100 | 100 | 100 | 100 | 100 | 100 | 98.74 | 98.74 |
| 016669 | atpA | ATP synthase F1, ß subunit | 95.14 | 99.61 | 99.61 | 100 | 99.61 | 99.81 | 99.61 | 98.64 | 98.44 |
| 005826 | pgk | Phosphoglycerate kinase | 93.02 | 100 | 100 | 98.97 | 100 | 99.74 | 99.74 | 97.67 | 97.83 |
| 009539 | tig | Trigger factor | 83.91 | 100 | 99.27 | 99.54 | 100 | 97.9 | 99.77 | 95.19 | 95.98 |
| 016619 | thdF/trmE | GTP binding, tRNA modification | 85.53 | 99.78 | 99.56 | 98.78 | 99.78 | 98.25 | 99.78 | 96.93 | 97.56 |

**Table S5.** Occurrence of different dioxygenases encoded in the *P.putida* LS46 genome and their homologues encoded in the genomes of other *P. putida* strains.

| **Locus Tag** | **Product Name** | **BIRD-1** | **DOT** | **F1** | **GB1** | **KT2440** | **LS46** | **ND6** | **S16** | **UW4** | **W619** |
| --- | --- | --- | --- | --- | --- | --- | --- | --- | --- | --- | --- |
| PPUTLS46_000020 | extradiol ring-cleavage dioxygenase III subunit | 1 | 1 | 1 | 1 | 1 | 1 | 1 | 1 | 0 | 1 |
| PPUTLS46_004082 | bifunctional nitric oxide dioxygenase/dihydropteridine reductase 2 | 1 | 1 | 1 | 1 | 1 | 1 | 1 | 1 | 0 | 1 |
| PPUTLS46_007854 | benzoate dioxygenase, alpha subunit | 1 | 1 | 1 | 1 | 1 | 1 | 1 | 1 | 1 | 1 |
| PPUTLS46_007854 | benzoate 1,2-dioxygenase | 1 | 1 | 1 | 1 | 1 | 1 | 1 | 1 | 1 | 1 |
| PPUTLS46_007859 | catechol 1,2-dioxygenase | 1 | 1 | 1 | 1 | 1 | 1 | 1 | 1 | 0 | 1 |
| PPUTLS46_007879 | glyoxalase/bleomycin resistance protein/dioxygenase | 0 | 0 | 0 | 0 | 0 | 1 | 0 | 0 | 0 | 0 |
| PPUTLS46_008999 | ring-cleaving dioxygenase | 1 | 1 | 1 | 0 | 1 | 1 | 1 | 0 | 0 | 0 |
| PPUTLS46_009209 | protocatechuate 4,5-dioxygenase | 1 | 1 | 1 | 0 | 1 | 1 | 1 | 0 | 0 | 1 |
| PPUTLS46_010694 | 4-hydroxyphenylpyruvate dioxygenase | 1 | 1 | 1 | 1 | 1 | 1 | 1 | 1 | 1 | 1 |
| PPUTLS46_010869 | 2-nitropropane dioxygenase, NPD | 1 | 1 | 1 | 1 | 1 | 1 | 1 | 1 | 0 | 1 |
| PPUTLS46_011160 | glyoxalase/bleomycin resistance protein/dioxygenase | 1 | 1 | 1 | 1 | 1 | 1 | 1 | 1 | 0 | 0 |
| PPUTLS46_012085 | protocatechuate 3,4-dioxygenase subunit beta | 1 | 1 | 1 | 1 | 1 | 1 | 1 | 1 | 0 | 1 |
| PPUTLS46_012893 | protocatechuate 3,4-dioxygenase subunit alpha | 1 | 1 | 1 | 1 | 1 | 1 | 1 | 1 | 0 | 1 |
| PPUTLS46_012898 | homogentisate 1,2-dioxygenase | 1 | 1 | 1 | 1 | 1 | 1 | 1 | 1 | 1 | 1 |
| PPUTLS46_013078 | Phytanoyl-CoA dioxygenase | 1 | 0 | 1 | 0 | 0 | 1 | 0 | 0 | 0 | 0 |
| PPUTLS46_015589 | Aromatic ring-cleaving dioxygenase | 1 | 1 | 1 | 1 | 1 | 1 | 1 | 1 | 0 | 1 |
| PPUTLS46_016409 | alpha-ketoglutarate-dependent dioxygenase AlkB | 1 | 1 | 1 | 1 | 1 | 1 | 1 | 1 | 0 | 1 |
| PPUTLS46_018151 | 4-hydroxyphenylpyruvate dioxygenase | 1 | 1 | 1 | 1 | 1 | 1 | 1 | 1 | 1 | 1 |
| PPUTLS46_018316 | 2-nitropropane dioxygenase | 1 | 1 | 1 | 1 | 1 | 1 | 1 | 1 | 1 | 1 |
| PPUTLS46_024143 | taurine dioxygenase | 1 | 1 | 1 | 1 | 1 | 1 | 1 | 1 | 0 | 1 |
| PPUTLS46_025213 | taurine dioxygenase | 0 | 0 | 1 | 1 | 1 | 1 | 1 | 1 | 1 | 1 |
| PPUTLS46_025478 | catechol 1,2-dioxygenase | 1 | 1 | 1 | 1 | 1 | 1 | 1 | 1 | 0 | 0 |
| Total |  | 20 | 19 | 21 | 18 | 20 | 22 | 20 | 18 | 7 | 17 |

**Table S6.** Presence of genes encoded in the *P. putida* KT2440 genome associated with different metabolic pathways in other *P.putida* strains.

| **PP_** | **Gene** | **Product** | **LS46** | **F1** | **GB1** | **BIRD-1** | **ND6** | **W619** | **S16** | **DOT** | **UW4** |
| --- | --- | --- | --- | --- | --- | --- | --- | --- | --- | --- | --- |
| 5120 | - | Aldehyde dehydogenase | 005051(97.9) | 4994 | 5770 | 4910 | 04372 | 0345 | 4965 | 4680 | 05143 |
| 1478 | - | Xenobioc reductase | 014259(95.1) | 4243 | 1086 | 4079 | 02692 | 4139 | 1130 | 2856 | 04419 |
| 1254 | xenA | Xenobiotic reductase (putative) | 021346(99.2) | 1281 | 4137 | 1290 | 07690 | 3913 | 0749 | 3152 | - |
| 3537 | pcbA | 4 hydroxy benzoate -3-monooxygenase | 002317(98.9) | 2237 | 2383 | 2257 | 00076 | - | 3029 | 0022 | 04363 |
| 2704 | - | Amidohydrolase family | 019116(98.7) | 3048 | 3083 | 2969 | 10879 | 2995 | - | 5481 | - |
| 3622 | - | Isoquinoline-1-oxidoreductase ß subunit | 025746(99.4) | 2111 | 2295 | 2166 | 09571 | 2834 | 3111 | 1805 | - |
| 3821 | galU | UTP-glucose-1-phosphate uridyl transferase | 011130(98.9) | 1949 | 3536 | 1959 | - | 3189 | 3303 | 1483 | 2411 |
| 2478 | - | Isoquinoline-1-oxidoreductase ß subunit | - | 2111 | 2296 | 2166 | - | 2833 | 3111 | 1806 | - |
| 2477 | - | Isoquinoline-1-oxidoreductase ∞ subunit | - | 2112 | - | 2167 | 09572 | 2834 | 3110 | 1805 | - |
| 3354 | - | Acyl CoA dehydrogenase/ferulic acid transferase | - | 2404 | - | - | - | 2051 | - | - | - |
| 3358 | - | Enonyl CoA hydratase/transferuloyl CoA hydratase | - | 2400 | - | - | - | 2047 | - | - | - |
| 3942 | - | Maleate cis trans isomerase | 011670(100) | 1895 | 3577 | 1866 | 08971 | 2142 | 4060 | 5125 | - |
| 3352 | - | Aryl sufatase | - | 2406 | - | - | ND14 | 1986 | - | 5507 | 0164 |
| 5253 | - | Aryl esterase | 017579(99.2) | 5163 | - | 5047 | 04632 | 0220 | 5105 | 2762 | - |
| 4490 | pphA | Phenylalanine-4-hydroxylase | 020721(100) | 1424 | 3995 | 1411 | 07921 | 3779 | 3835 | 4057 | 3913 |
| 3282 | paaC | 3 hydroxybutaryl CoA dehydrogenase | 008344(96.8) | 2477 | 2614 | 2490 | 10793 | 2627 | 2689 | 5591 | - |
| 3270 | paaN | oxidoreductase | 008284(99.4) | 2489 | 2626 | **2502** | **010772** | 2639 | 2701 | 5603 | - |
| 5006 | phaD | Transcriptional regulator | 005606(99.0) | 4880 | 5056 | 4794 | 04112 | - | 4850 | 5661 | 00330 |
| 5003 | phaA | PHA synthase | 005611(99.3) | 4877 | 5053 | 4791 | 04108 | 0458- | 4897 | 5658 | 00333 |
| 2803 | dhaT | Propanediol dehydrogenase | - | - | - | - | - |  | - | - | - |
| 4656 | pcaH | Protocatechuate 3,4 dioxygenase ß subunit | 012893 (99.5) | 4518 | 4651 | 4352 | 03247 | 0780 | 4486 | 0830 | - |
| 4655 | pcaG | Protocatechuate 3,4 dioxygenase ∞ subunit | 012898(98.1) | 4517 | 4650 | 4351 | 03245 | 0781 | 4485 | 0829 | - |
| 3952 | pcaJ | 3-oxoadipate CoA transferase ß subunit | 011720(100) | 1870 | 3594 | 0054 | 08918 | 2596 | 1841 | 2058 | - |
| 3951 | pcaJ | 3-oxoadipate CoA transferase ∞ subunit | 011715(100) | 1871 | 3593 | 0053 | 08920 | 2597 | 1840 | 2059 | - |
| 1381 | pcaC | 4-carboxymuconolactone decarboxylase | 013753(99.3) | 4342 | 4433 | 4179 | 02898 | 1021 | 4272 | 0238 | 1208 |
| 1379 | pcab | 3-carboxycis-cis muconatecycloisomrase | 013743(100) | 4344 | 4435 | 4181 | 02900 | 1020 | 4274 | 0236 | 1206 |
| 3569 | tauD | Taurine metabolism | 002142(99.0) |  | 2352 | 2225 | 00152 | 2956 | 3064 | 2295 | - |
| 0230 | tauD | Taurine metabolism | 025213(98.6) | 0245 | 0254 | 0256 | 05448 | 4982 | 0223 | 4628 | 00197 |
| 0169 | tauD | Taurine metabolism | 025478(99.0) | 0190 | 0188 | - | 05336 | - | 0141 | - | 00118 |
| 0920 | xenB | TNT metabolism | 022986(99.4) | 0959 | 4468 | 0971 | 06941 | 0986 | 4309- | 4758 | 01216 |
| 3357 | vdh | Vanillin degradation | 025298(34.8) | 2401 | - | - | - | 2048 | - | - | - |
| 3737 | vanB | Vanillate oxidoreductase | 026161(97.8) | 2026 | 2169 | 2025 | 9406 | 2044 | 9406 | 1716 | - |
| 3736 | vanA | Vanillate demethylase | 026156(98.6) | 2027 | 2170 | 2026 | 9409 | 2045 | 9409 | 1717 | - |
| 4753 |  | N-methyl proline demethylase | 024748(42.1) | 0331 | 0333 | 4459 | 05623 | 4897 | 5623 | 2558 | 5025 |
| 4752 |  | Aminopeptidase | - |  | - | 4458 | - | - | 2304 | - | - |

**Table S7.** Presence of genes encoded by the *P. putida* F1 genome involved in aromatic compound degradation and their homologues encoded in the genomes of other *P. putida* strains.

| ***P. putida* F1** | **Gene** | | **Product** | **LS46** | **W619** | **KT2440** | **GB1** | **BIRD1** | **ND6** | **S16** | **DOT** | **UW4** |
| --- | --- | --- | --- | --- | --- | --- | --- | --- | --- | --- | --- | --- |
| Pput_ |  | |  | PPUT  LS46_ | Pput  W619_ | PP_ | Pput  GB1_ | Pput  BIRD1_ | YSA_ | PPS_ | TIE_ | Pput  UW4_ |
| 2867 | *sepA*  *ttgA* | | RND family effux transporter | 013778 | 1026 | 1386 | 4427 | 4174 | 02885 | 4267 | 0243 | 1213 |
| 2868 | *sepB*  *ttgB* | | Hydrophobe/amphophile efflux | 013773 | 1025 | 1385 | 4928 | 4175 | 02888 | 4268 | 0242  4280 | 1212 |
| 2869 | *sepC*  *ttgC* | | RND efflux outer membrane lipoprotein | 013768 | 1024 | 1384 | 4429 | 4176 | 02890 | 4269 | 0241  4279 | 1211 |
| 2871 | *todT* | | Response regulator | - | - | - | - | - | - | - | 4277 | - |
| 2872 | *todS* | | Signal transduction histidine kinase | - | - | - | - | - | - | - | 4275  4276 |  |
| 2876 | *todE* | | 3-methylcatechol 2,3 dioxygenase | - | - | - | - | - | - | - | 4271 |  |
| 2877 | *todD* | | Cis toluene dihydrodiol dehydrogenase | - | - | - | - | - | - | - | 4270 |  |
| 2878 | *todA* | | Aromatic ring hydroxylating dioxygenase | - | - | - | - | - | - | - | 4269 |  |
| 2879 | *todB* | | Ferridoxin | - | - | - | - | - | - | - | 4268 |  |
| 2880 | *todC2* | | Toulene dioxygenase | - | - | - | - | - | - | - | 4267 |  |
| 2881 | *todC1* | | Toulene dioxygenase | - | - | - | - | - | - | - | 4266 |  |
| 2882 | *todF* | | 2-hydroxy-6-oxo-2,4-heptadiennoate hydrolase | - | - | - | - | - | - | - | 4265 |  |
| 2883 | *todX* | | Membrane protein | - | - | - | - | - | - | - | 4262 |  |
| 2887 | *-* | | Enoy-coenzyme A hydratase | - | - | - | - | - | - | - | 4259 |  |
| - | *mhpT* | | 3-hydrophenylpropionic acid transporter | - | - | - | - | - | - | - |  |  |
| 2888 | *cmtG*  *mphE* | | 4-hydroxy-2-oxovalerate aldolase | - | - | - | - | - | - | - | 4258 |  |
| 2889 | *cmtH*  *mphD* | | Acetaldehyde dehydrogenase | - | - | - | - | - | - | - | 4257 |  |
| 2890 | *cmtF*  *mphD* | | 2-Hydroxypenta-2-,4-dienoate hydratase | - | - | - | - | - | - | - | 4256 |  |
| 2891 | *cmtE* | | HOMODA hydrolase | - | - | - | - | - | - | - | 4255 |  |
| 2892 | *cmtI* | | Protein of unknown function | - | - | - | - | - | - | - | 4254 |  |
| 2893 | *cmtD* | | HCOMODA | - | - | - | - | - | - | - | 4253 |  |
| 2894 | *cmtAD* | | p-cumate dioxygenase ferridoxin subunit | - | - | - | - | - | - | - | 4252 |  |
| 2895 | *cmtB* | | 2,3-dihydroxy-2,3 dihydro-p-cumate dehydrogenase | - | - | - | - | - | - | - | 4251 |  |
| 2896 | *cmtC* | | 2,3-dihydroxy-p-cumate-3,4=dioxygenase | - | - | - | - | - | - | - | 4250 |  |
| 2897 | *cmtAc* | | p-Cumate dioxygenase small subunit | - | - | - | - | - | - | - | 4249 |  |
| 2898 | *cmtAb* | | p-Cumate dioxygenase large subunit | - | - | - | - | - | - | - | 4248 |  |
| 2899 | *cmtAa* | | p-cumate dioxygenaseferridoxin reductase subunit | - | - | - | - | - | - | - | 4247 |  |
| 2900 | *cymE* | | Acetyl CoA synthetase | - | - | - | - | - | - | - | 4246 |  |
| 2901 | *cymD* | | Outer membrane protein | - | - | - | - | - | - | - | 4245 |  |
| 2902 | *cymAb* | | p-Cymene monooxygenase reductase unit | - | - | - | - | - | - | - | 4244 |  |
| 2903 | *cymAa* | | p-Cymene monooxygenase | - | - | - | - | - | - | - | 4243 |  |
| 2904 | *cymC* | | p-Cumic aldehyde dehydrogenase | - | - | - | - | - | - | - | 4242 |  |
| 2905 | *cymB* | | p-Cumic alcohal dehydrogenase | - | - | - | - | - | - | - | 4241 |  |
| 2906 | *cymR* | | Reglatory protein for cym and cmt operon | - | - | - | - | - | - | - | 4240 |  |
| ***Nicotinic acid and nictinamide degradation genes*** | | | | | | | | | | | | |
| **Locus tag** | ***gene*** | | **Product** | **LS46** | **W619** | **GB-1** | **BIRD-1** | **ND6** | **S16** | **DOT** | **F1** | **UW4** |
| PP_3939 | *nicP* | | Benzoate porin | 11655 | 2145 | 3574 | 1869 | 08976 | 4075 | 5129 | 1898 | 2654 |
| PP_3940 | *nicT* | | Tartrate transporter | 11660 | 2144 | 3575 | 1868 | 08975 | 4595 | 5127 | 1897 | 2655 |
| PP_3941 | *nicF* | | Nicotinamidase | 11665 | 2143 | 3576 | 1867 | 08973 | 4057  (36.6)* | 5126 | 1896 | 2656 |
| PP_3942 | *nice* | | Maleate cis-trans isomerase | 11670 | 2142 | 3577 | 1866 | 08971 | 4060  (69.3)* | 5125 | 1895 | 3242 |
| PP_3943 | *nicD* | | ∞ß hydrolase deformylase | 11675 | 2141 | 3578 | 1865 | 08969 | 4059  (55.6)* | 5124 | 1894 | 2747 |
| PP_3944 | *nicC* | | 6 hydroxynicotinic acid 3 monooxygenase | 11680 | 2140 | 3579 | 1864 | 08966 | - | 5123 | 1893 | - |
| PP_3945 | *nicX* | | Hypothetical protein | 11685 | 2139 | 3580 | 1863 | 08963 | 4058  (43.7)* | 5122 | 1892 | - |
| PP_3946 | *nicR* | | Multiple antibiotic resistance repressor | 11690 | 2138 | 3581 | 1862 | 08962 | - | 5121 | 1891 | 3765 |
| PP_3947 | *nicA* | | Isoquinoline 1 oxidoreductase (subunit A) | 11695 | 2137 | 3582 | 1861 | 08961 | - | - | 1890 | 2894 |
| PP_3948 | *nicB* | | Isoquinoline 1 oxidoreductase (subunit B) | 11700 | 2136 | 3583 |  | 08959 | - | - | 1889 | 2625 |
|  |  | |  | * percentage aa sequence homology to *P.putida* LS46 proteins | | | | | | | | |
| ***Manganese oxidation*** | | | | | | | | | | | | |
| **Locus PputGB1_** | ***Gene*** | | **Product** | **LS46** | **F1** | **KT2440** | **W619** | **BIRD** | **ND6** | **S16** | **DOT** | **UW4** |
| 2447 | *mnxG* | | Mn (II) oxidase | 002532 | 2456 | 3490 | 2456 | 2023 | 11343 | 2966 | 01642 | 2456 |
| 2665 | *mnxG* | | Mn (II) oxidase | 006964 | 2532 | 3184 | - | - | 10685 | 2743 | 01039 | 1904 |
| ***Siderophore*** | | | | | | | | | | | | |
|  | *pvdE* | | Pyoverdine transporter | 012775 | 1681 | 4216 | 4089 | 1634 | 08482 | 3925 | 1278 | 1567 |
|  | *fpvA* | | Ton dependent receptor gene | - | - | - | - | - | - | - | - | - |
| ***Oxidative stress*** | | | | | | | | | | | | |
| PputW619_4269 | | *sodA* | Superoxide dismutase | 022846 | 0985 | 0946 | 0933 | 0999 | 06985 | 0975 | 05070 | - |
| 0981 | | *sodB* | Superoxide dismutase | 023011 | 0954 | 0915 | 4473 | 0966 | 06924 | 4314 | 01925 | 04371 |
| 2485 | | *sodC* | Superoxide dismutase | - | - | - | - | - | - | - | - | - |
| 4722 | | *katA* | Catalase | 023883 | 0514 | 0481 | 0510 | 0518 | 05979 | 0477 | 03479 | 4857 |
| 2032 | | *katB* | Catalase | 016234 | 0132 | 0115 | 0130 | 0143 | 05195 | 0079 | 04765 | 1756 |
| 5113 | | *katE* | Catalase | - | - | - | - | - | - | - | - | - |
| 2235 | | *katG* | Catalase | 026006 | 2061 | 3668 | - | 2060 | 09477 | - | 01753 | 3185 |
| 2390 | |  | Catalase | 020296 | 2803 | 2887 | 2895 | 2848 | 09997 | 2552 | 01657 | 1756 |
| 3186 | | *ahpF* | Alkylhydroperoxide reductase | 010259 | 3255 | 2440 | 3533 | 3242 | 00964 | 3300 | 05238 | 2417 |
| 3187 | | *ahpC* | Alkylhydroperoxide reductase | 010254 | 3256 | 2439 | 3534 | 3243 | 00966 | 3301 | 05239 | 2416 |
| 1113 | | *ahpC* | Alkylhydroperoxide reductase | 022151 | 1125 | 1084 | 4328 | 1134 | 07248 | 4171 | 4718 | 04406 |
| 3104 | | *ahpD* | Alkylhydroperoxide reductase | 026236 | 2011 | - | 2151 | 2010 | 09380 | 3222 | 0147 | 02205 |
| 3108 | | *ahpD* | Alkylhydroperoxide reductase | 010169 | 2713 | - | 2151 | 3265 | 09539 | 2266 | 5257 | 02835 |
| 3238 | | *ahpD* | Alkylhydroperoxide reductase | 003162 | 2094 | 2959 | 2062 | 2708 | 10192 | 3132 | 3850 | 01589 |
| 1849 | |  | Cloroperoxidase | 025028 | 0281 | 0266 | 2273 | 0293 | 05510 | 0259 | 2503 | 00235 |
| 2803 | |  | Thiol peroxidase | 021426 | 2185 | 3587 | 4623 | 2206 | 00198 | 3081 | 2275 | 02814 |
| 3977 | |  | Thiol peroxidase | 021426 | 1265 | 1235 | 4626 | 1273 | 07556 | 3982 | 5031 | 01285 |
| 1244 | |  | Glutthioneperoxidase | 014993 | 4033 | 1686 | 2328 | 3931 | 02414 | 1337 | 3636 | 03762 |
| 1483 | |  | Glutathione peroxidase | 000039 | 3841 | 1874 | 4183 | 3741 | 02010 | 1511 | 1488 | 01443 |
| 3188 | |  | Glutaredoxin oxidoreductase | 026491 | 1950 | 3819 | 1284 | 1960 | 09269 | 3302 | 0339 | 02413 |
| 3239 | |  | Glutaredoxin | 003157 | 2714 | 2958 | 5105 | 2709 | 10191 | 2267 | 3849 | 00278 |
| 1469 | | *ohr* | Organic oxidoredutase protein | 015954 | 3855 | 1859 | 1434 | 3755 | 02037 | 1496 | 5087 | 01427 |
| 1470 | | *ohrR* | Organic oxidoredutase resistance transcriptional regulator | 015959 | 3854 | 1860 | 1435 | 3754 | 02034 | 1497 | 5086 | 01428 |
| 4378 | | *nor* | Nitric oxide dioxygenase |  |  |  |  |  |  |  |  |  |
| 4379 | | *norR* | Nitric oxide reductase transcriptional regulator | 009109 | 0831 | 0807 | 0846 | 0853 | 06693 | 0861 | 4589 | 00848 |
| 2615 | | *perR* | Peroxide resistant protein | - | - | - | - | - | - | - | - | - |
| 4005 | |  | DNA binging stress protein | 021566 | 1239 | 1210 | 4007 | 1247 | 07503 | 4007 | 2954 | - |
| 0702 | | *fur* | Ferric uptake regulator | 007086 | 4596 | 4070 | 4576 | 4433 | 03430 | 4576 | 0658 | 00687 |
| 4721 | | *bfrA* | Bacterioferritin œ subunit | 023878 | 0515 | 0482 | 0478 | 0519 | 05981 | 0478 | 3480 | 04856 |
| 1111 | | *bfrB* | Bacterioferritin | 022166 | 1123 | 1082 | 4173 | 1132 | 07242 | 4173 | 4720 | 04408 |
| 1112 | | *Bfd* | Bfd associated protein | 022161 | 1124 | 1083 | 4172 | 1133 | 07246 | 4172 | 4719 | 04407 |
| 1113 | | *ahpC* | Alkylhydroperoxide reductase | 022151 | 1125 | 1084 | 4171 | 1134 | 07248 | 4173 | 4718 | 04406 |
| 4791 | | *acnA* | Hydroperxide resistant Aconitase | 024238 | 0445 | 0411 | 0407 | 0449 | 058331 | 0407 | 2142 | 04928 |
| 4269 | | *fumC* | Hydroperoxide resitant fumarase | 022846 | 0985 | 0946 | 0975 | 0999 | 06985 | 0975 | 5070 | - |
| **Pseudomonine** | |  | | | | | | | | | | |
| PSEEN2500-2507 | |  | Pseudomonine synthesis genes | ND | ND | ND | ND | ND | ND | ND | ND | ND |

**Table S8.** Occurrence of heavy metal tolerance genes encoded by *P. putida* W619 in different *P. putida* strains.

| **Pputw619_** | **Gene** | **Product** | **LS46** | **F1** | **KT2440** | **GB1** | **BIRD-1** | **ND6** | **S16** | **DOT-T1E** | **UW4** |
| --- | --- | --- | --- | --- | --- | --- | --- | --- | --- | --- | --- |
|  |  |  | **PputLS46_** | **Pput_** | **PP_** | **PputGB1_** | **PputBIRD1_** | **YSA_** | **PPS_** | **TIE_** | **PputUW4_** |
| 0011(1) | copG | Survival in the presence of high bioavailable Cu (II) | 004439  008579 | 0011 | 5377 | 0013 | 3448 | 1377 | 5264  1814 | 4740 | 02449 |
| 0012 (1) | copM | Cytochrome C family protein | 023593  008584  008579 | 0012 | 5378 | 0014 | - | 1377  6086 | 5363 | 5752  4740 | - |
| 0013 (1) | copB | Copper resistant protein B | 023588 | 0013 | 5379 | 0015 | - | - | 5262 | 5753 | - |
| 0014(1) | copB | Putative copB | - | 0014 | - | 0016 | - | - | 5261 | - | - |
| 0015(1) | copA | Copper resistant protein A | 008599  023578 | 0015 | 5380 | 0017 | - | 6093 | 0532  5260  5755 | 4513 | - |
| 0017(1) | copR | Transcriptional activator | 008669 | 0017 | 5383 | 0019 | - | - | 5258 | - | - |
| 0018 (1) | copS | Sensor protein | 008674 | 0018 | 5384 | 0020 | - | - | 5257 | - | - |
| 0020(1) | cusC | Cu/Ag tricomponent efflux outer menbrane porin | - | 0020 | 5385 | 0022  3041 | - |  | 5285 | - | - |
| 0021(1) | cusB | Cu/Ag tricomponent efflux membrane fusion protein | - | 0021 | 5386 | 0023  3041 | - | - | 5254 | - | - |
| 0022 (1) | cusA | Copper transporter RND family | 008564 | 0022 | 5387 | 0024  3043 | - | - | 5253 | - | - |
| 0023 (1) | cusF | Periplasmic copper binding protein | - | 0023 | 5388 | 0025 | - | - | - | - | - |
| 0024 (1) |  | S-isopprenylcysteine methyl transferase (czcN homolog) | - | 0025 | 5389 | 0026 | - | - | - | - | - |
| 0029(1) | copF | Copper P-type ATPase | 008644 | 0030 | - | 0031 | - | - | - | - | - |
| 0043 (1) | czcD | Cation diffusion faciltator (CDF) | 017334 | 0040 | - | 0040 | - | 04743 | 5227 | - | - |
| 0046(1) | czcR | DNA binding response regulator | 017319 | 0043 | 0029 | 0043 | - | 04750 | 5240 | 2811 | - |
| 0047 (1) | czcS | Sensory Histidine kinase | 017314 | 0044 | 0030 | 0044 | - | 04754 | 5241 | 2812 | - |
| 0050 (1) | gtrM | Glycosyltransferase (protein of O-glycosylation) | 017299 | 0047 | 0033 | 0047 | - | 04760 | 5244 | 2815 | - |
| 0051(1) | gtrB | Glycosyltransferase (bactoprenol) | 017294 | 0048 | 0034 | 0048 |  | 04761 | - | 2816 | - |
| 0052(1) | gtrA | Bactoprenol –linkedglucose translocase | - | 0049 | 0035 | 0049 | - | - | - | - | - |
| 0058 (1) | cadA | Cadmium translocating P-type ATPase | - | 055 | 0041 | 0055 | - | - | - | - | - |
| 0060 (1) | czcA | Cobalt/zinc/cadmium efflux RND transporter | 008634  010104  017249 | 0057 | 0043 | 0057 | - | 04784  01021 | 5247  1997 | 5270  4694 | - |
| 0061 (1) | czcB | Cobalt/zinc/cadmium efflux RND transporter | 010099  017244 | 0058 | 0044 | 0058 | - | 04786 | 5248  1996 | 5271  4695 | - |
| 0062 (1) | czcC | Cobalt/zinc/cadmium resitant protein | 017239 | 0059 | 0045 | 0059 | - | 04789 | 5249  1995 | 4696 | - |
| 0063 (1) | - | Putative porin OprD family | 017229 | 0060 | 0046 | 0060 | - | 4790 | 5250 | 4697 | - |
| 0064 (1) | czcR | DNA binding heavy metal response regulator | 017234 | 0061 | 0047 | 0061 | - | 04795 | 5251 | 4698 |  |
| 0325 | cadR | Transcriptional regulator | 004946 | 5013 | 5140 | 5193 | 4931 | 04411 | 4988 | 4488 | 5157 |
| 0326 | cadA | Cadmium translocating P-type ATPase | 004951 | 5012 | 5139 | 5192 | 4931 | 4408 | 4987 | 4489 | 5166 |
| 1207 | arsC | Arsenate reductase | 014799 | 4072 | 1645 | 1247 | 3971 | 02486 | 1298 | 4996 | 4117 |
| 1676 | cinA | Copper containing azurin like protein | 004514 | 3583 | 1463 | 1700 | 3495 | 01463 | - | 1234 | - |
| 1677 | cinQ | Pre Qo reductase | 004519 | 3494 | 1462 | 1701 | 3494 | 01462 | 1727 | - | 3498 |
| 1712 (8) | copB | Copper resistant protein B | 008579 | - | - | - | - | 06089 | 0530 | 5753 | - |
| 1713 (8) | copA | Copper resistant protein A | 004599 | - | - | - | 3446 | 06093 | 0532 | 5755 | 3484 |
| 1714 (8) | - | Putative zinc transporter | - | - | - | - | - | - | - | - | 1714 |
| 1715 (8) | arsB | Arsenate efflux pump | - | - | - | - | - | - | - | - | - |
| 2323 (11) | merR | Mercury resistant operon regulatory protein | - | - | - | - | - | - | - | - | - |
| 2324 (11) | merB | Alkylmercury lyase | - | - | - | - | - | - | - | - | - |
| 2325 (11) | merR | Transcriptional regulator MerR family | - | - | - | - | - | ND20 | - | - | - |
| 2326 (11) | - | Heavy metal/H + antiport, CDF family | - | - | - | - | - | ND21 | - | - | - |
| 2336 (11) | merE | Mercuric resistant protein | - | - | - | - | - |  | - | - | - |
| 2337 (11) | merD | HTH type transcriptional regulator | - | - | - | - | - | 03476 | 5233 | - | - |
| 2338(11) | merA | Mercuric Hg (II) reductase | - | - | - | - | - | 03475 | 5234 | - | - |
| 2339 (11) | merP | Mercuric transporter protein periplasmic component | - | - | - | - | - | 03473 | 5235 | - | - |
| 2340 (11) | merT | Mercuric transport protein | - | - | - | - | - | 03471 | 5236 | - | - |
| 2341 (11) | merR | Mercury resistant operon regulatory protein | - | - |  | - | - | 03469 | 5237 | - | - |
| 3004 (18) | nikR | Putative nickel response regulator | - | - | 3341 | - | - |  | - | - | - |
| 3005 (18) | nikA | Nickel ABC transporter, periplasmic nickel binding protein | - | - | 3342 | - | - |  | - | - | - |
| 3006 (18) | nikB | Nickel transporter permease NikB | - | - | 3343 | - | - |  | - | - | - |
| 3007 (18) | nikC | Nickel transporter permease NikC | - | - | 3344 | - | - |  | - | - | - |
| 3008 (18) | nikD | Nickel import ATP binding protein NikD | - | - | 3345 | - | - |  | - | - | - |
| 3009 (18) | nikE | Nickel import ATP binding protein NikE | - | - | 3346 | - | - |  | - | - | - |
| 3017 (18) | chrA | Chromate transporter | - | - | - | - | - |  | - | - | - |
| 3197 | modA | Mo ABC transporter, periplasmic Mo binding protein | 011165 | 3828 | 1952 | 3543 | 1952 | 09249 | 3310 | 1476 | 2399 |
| 3198 | modB | Mo ABC transporter permease protein | 011170 | 3829 | 1951 | 3544 | 1951 | 09248 | 3311 | 1475 | 2398 |
| 3199 | modC | Mo ABC transporter, ATP binding protein | 011175 | 3830 | 1950 | 3545 | 1950 | 09246 | 3312 | 1474 | 2397 |
| 4088 | arsC | Arsenate reductase | 014499 | 4193 | 1531 | 1140 | 4029 | 02592 | 1181 | 4791 | 1082 |
| 4576 | - | Putative copper binding protein | 023318 | 0627 | 0588 | 0633 | 0638 | 06197 | 0587 | 3759 | - |
| 4578 | - | Putative copper translocating protein | 023328 | 0625 | 0586 | 0631 | 0636 | 06195 | 0585 | 3757 | 0579 |
| 4579 | merE | Transcriptional regulator heavy metal dependent MerE family |  |  |  |  |  |  |  |  |  |
| 5108 | znuA | Zinc uptake ABC transporter, periplasmic ninding protein | 016209 | 0137 | 0129 | 0135 | 0148 | 05205 | 0084 | 4760 | 0067 |
| 5109 | zur | Transcriptional repressor of Zn transport system | 016214 | 0136 | 0119 | 0134 | 0147 | 05204 | 0083 | 4761 | 0066 |
| 5110 | znuC | Zinc ABC transporter, ATP binding protein | 016219 | 0135 | 0118 | 0133 | 0146 | 05202 | 0082 | 4762 | 0065 |
| 5111 | znuB | Zinc ABC transporter, permease protein | 016224 | 0134 | 0117 | 0132 | 0145 | 05201 | 0081 | 4763 | 0064 |
| 5146 (31) | arsR | Arsenical resistance operon repressor | 019041 | 3034 | 1930 | 3077 | 2954 | 00647 | 2281/5222 | 5223 | - |
| 5147 (31) | arsC | Arsenate reductase | 019051 | 3036 | 1928 | 3079 | 2956 | 00649 | 2280/5221 | 5220 | - |
| 5148 (31) | arsB | Arsenite efflux transporter | 019056 | 3035 | 1929 | 3078 | 2955 | 00652 | 2279/5229 | 5221 | - |
| 5149 (31) | arsH | Arsenical resistance protein | 019046 | 3037 | 1927 | 3080 | 2957 | 00654 | 2278/5219 | 5219 | - |
| 5156 (31) | chrF | Chromate resistant regulator | - | - | - | - | - | - | - | - | - |
| 5157 (31) | chrA | Chromate transporter | 010879 | 3159 | 2556 | 3384 | 3120 | 00454 | 2097 | 3354 | 3067 |
| 5158 (31) | chrB | Chromate resistance protein | - | - | - | - | - | - | - | - | - |
| 5159 (31) | nreB | Major facilitator superfamily (Nickel efflux family) | - | - | - | - | - | - | - | - | - |
| 5161 (31) | - | Putative cation diffusion facilitator (CDF) | - | - | - | - | - | - | - | - | - |
| 5177 (31) | copS | Sensor protein CopS | - | - | - | - | - | - | - | - | - |
| 5178 (31) | copR | Transcriptional regulator CopR | - | - | - | - | - | - | - | - | - |
| 5180 (31) | copA | Copper resistance protein CopA |  | 0574 | 2205 | 1828 |  |  |  |  |  |
| 5181 (31) | copB | Putative CopB (frameshifted) |  | - | - | - |  |  |  |  |  |
| 5182 (31) | copB | Copper resistance protein CopB |  | 0573 | 2204 | 1827 |  |  |  |  |  |
| 5183 (31) | copM | Cytochrome C family protein |  | 0572 | - | - |  |  |  |  |  |
| 5184 (31) | copG | Involved in survival in the presence of high bioavailable Cu(II) |  | - | 2203 | 1826 |  |  |  |  |  |

**Table S9.** RND transporter genes encoded in the *P. putida* LS46 genome and their homologues in the genomes of other *P. putida* strains.

| **Locus Tag** | **Product Name** | **BIRD-1** | **DOT-T1E** | **F1** | **GB1** | **KT2440** | **LS46** | **ND6** | **S16** | **UW4** | **W619** |
| --- | --- | --- | --- | --- | --- | --- | --- | --- | --- | --- | --- |
| PPUTLS46_000550 | RND efflux transporter | 1 | 1 | 1 | 1 | 1 | 1 | 1 | 1 | 1 | 1 |
| PPUTLS46_000765 | RND family efflux transporter MFP subunit | 1 | 1 | 1 | 1 | 1 | 1 | 1 | 0 | 0 | 0 |
| PPUTLS46_000790 | RND efflux system outer membrane lipoprotein | 1 | 1 | 1 | 0 | 1 | 1 | 1 | 0 | 0 | 0 |
| PPUTLS46_001302 | RND efflux system outer membrane lipoprotein | 1 | 1 | 1 | 1 | 1 | 1 | 1 | 1 | 0 | 1 |
| PPUTLS46_002047 | RND family efflux transporter MFP subunit | 1 | 1 | 1 | 1 | 1 | 1 | 1 | 1 | 0 | 1 |
| PPUTLS46_002062 | RND efflux system outer membrane lipoprotein | 1 | 1 | 1 | 1 | 1 | 1 | 1 | 1 | 0 | 1 |
| PPUTLS46_004771 | Efflux transporter, RND family, MFP subunit | 1 | 1 | 1 | 1 | 1 | 2 | 1 | 1 | 0 | 1 |
| PPUTLS46_004776 | RND family efflux transporter MFP subunit | 1 | 1 | 1 | 1 | 1 | 1 | 1 | 1 | 0 | 1 |
| PPUTLS46_008559 | RND family efflux transporter MFP subunit | 0 | 0 | 0 | 0 | 0 | 1 | 0 | 0 | 0 | 0 |
| PPUTLS46_009134 | RND efflux system, outer membrane lipoprotein | 0 | 1 | 0 | 0 | 0 | 1 | 0 | 0 | 0 | 0 |
| PPUTLS46_010354 | Rnd efflux pump, membrane fusion protein, czcb subfamily | 0 | 0 | 0 | 0 | 0 | 1 | 0 | 0 | 0 | 0 |
| PPUTLS46_012755 | RND family efflux transporter MFP subunit | 0 | 1 | 1 | 0 | 0 | 1 | 1 | 0 | 1 | 0 |
| PPUTLS46_012765 | RND efflux system outer membrane lipoprotein | 0 | 1 | 1 | 0 | 0 | 1 | 1 | 0 | 0 | 0 |
| PPUTLS46_013183 | RND efflux system outer membrane lipoprotein | 1 | 1 | 1 | 1 | 1 | 1 | 1 | 1 | 0 | 1 |
| PPUTLS46_013223 | RND efflux system outer membrane lipoprotein | 1 | 1 | 1 | 1 | 1 | 1 | 1 | 1 | 0 | 1 |
| PPUTLS46_013778 | RND family efflux transporter MFP subunit | 1 | 1 | 1 | 1 | 1 | 1 | 1 | 1 | 0 | 1 |
| PPUTLS46_014429 | RND family efflux transporter MFP subunit | 1 | 1 | 1 | 1 | 1 | 1 | 1 | 1 | 0 | 1 |
| PPUTLS46_016734 | RND efflux transporter | 0 | 0 | 0 | 0 | 0 | 1 | 0 | 0 | 0 | 0 |
| PPUTLS46_018114 | RND family efflux transporter MFP subunit | 1 | 1 | 1 | 1 | 1 | 2 | 1 | 1 | 0 | 1 |
| PPUTLS46_018276 | Efflux transporter RND family, MFP subunit | 1 | 1 | 1 | 1 | 1 | 1 | 1 | 1 | 0 | 1 |
| PPUTLS46_018286 | RND efflux system outer membrane lipoprotein | 1 | 2 | 1 | 1 | 1 | 1 | 1 | 1 | 0 | 1 |
| PPUTLS46_018391 | RND family efflux transporter MFP subunit | 1 | 1 | 1 | 1 | 1 | 1 | 1 | 1 | 0 | 1 |
| PPUTLS46_023071 | RND family efflux transporter MFP subunit | 1 | 1 | 1 | 0 | 1 | 1 | 1 | 1 | 0 | 1 |
| PPUTLS46_025428 | RND efflux transporter | 1 | 1 | 1 | 1 | 1 | 1 | 1 | 1 | 0 | 1 |
| Total |  | 18 | 22 | 20 | 16 | 18 | 26 | 20 | 16 | 2 | 16 |
